# Even–Odd Layer Oscillatory Behavior of Electronic and Phononic Specific Heat in an Ultra-Thin Metal Film

**DOI:** 10.3390/ma17194851

**Published:** 2024-10-01

**Authors:** Shiyao Chong, Jian-Qi Shen

**Affiliations:** 1 College of Electronics and Information Engineering, Hengshui University, Hengshui 053000, China; 3110104339@zju.edu.cn; 2 Centre for Optical and Electromagnetic Research, College of Optical Science and Engineering, State Key Laboratory of Extreme Photonics and Instrumentation, East Building No. 5, Zijingang Campus, Zhejiang University, Hangzhou 310058, China

**Keywords:** quantum size effect, electronic and phononic specific heat, ultra-thin metal film, electronic Fermi wavelength, phononic Debye frequency

## Abstract

Both electronic and phononic statistical and thermal properties, modulated by the quantum size effect, are suggested in a thin metal film. In order to show the quantum size effect of specific heat, the densities of the electron and phonon states of an ultra-thin film are treated within the framework of quantum statistics. It was found that strong and weak “*even–odd layer oscillatory behavior*” was exhibited by the ultra-thin metal film in electronic and lattice specific heat, respectively. Such a behavior, which depends on film thickness, results from the quantum confinement of electrons and phonons in the vertical (thickness) direction of the film, where both electrons and phonons form their respective quantum well standing wave modes. If, for example, the thickness of the ultra-thin metal film is exactly an integer multiple of a half wavelength of the standing wave of electrons in the thickness direction, the corresponding density of states would become maximized, and the electronic specific heat would take its maximum. In the literature, less attention has been paid to the size-dependent electron Fermi wavelength for quantum size effects, i.e., the Fermi wavelength in ultra-thin metal films has always been identified as a constant. We shall show how the Fermi wavelength varies with the size of a nanofilm, including an explicit analytic formulation for the thickness dependence of the electron Fermi wavelength. Size-dependent resonantly oscillatory behavior, depending on the ultra-thin or nanoscale film thickness, would have possible significance for researching some fundamental physical characteristics (e.g., low-dimensional quantum statistics) and may find potential applications in new thermodynamic device design.

## 1. Introduction

With the development of film technology [1,2], a variety of thin films have been utilized in optoelectronic and photonic device design, e.g., optical coatings [3], thin film photovoltaic cells [4,5], and many metallic or semiconductor electronic functional devices [2,6], as well as in surface plasmonic applications [7,8,9,10]. Ultra-thin films can have new interesting properties. Specifically, if, for example, the geometric size and shape of a material is limited in one or two dimensions, some physical characteristics related to the density of the states of electrons will be completely different to those of bulk materials, because in the thickness direction of the thin film, there are standing wave modes of electron de Broglie matter that are wave-modulated by the geometric size and shape of the nanostructures of the materials. This effect plays a key role in quantum wells as well as quantum wires and quantum dots. These quantum well eigenstates of electrons are quite sensitive to the thickness of ultra-thin films, as a stationary standing wave eigenstate of electrons in a quantum well should obey the constraint that the thin film thickness is an integral multiple of a half wavelength of an electron matter wave. Thus, the density distribution of electron states near the Fermi surface depends strongly on the thin film thickness; hence, this would have a substantial influence on many physical properties of a thin film [11,12], including the optical, electronic, superconducting and thermodynamic characteristics or performance. A nanoscale film is a layer of ultra-thin material with a thickness ranging from only several Ångstroms to dozens (or hundreds) of nanometers. Some measurable quantities of these thin films can exhibit size-dependent oscillatory behavior (quantum size effects [12,13,14,15]) when the film thickness is increased by film techniques, e.g., physical deposition or epitaxial growth. Such measurable physical parameters, which depend on nanofilm thickness, include the superconducting transition temperature [12], the electric conductivity [13,14], the metallic work function [15], magnetoresistance [13], the Hall constant [13,16], the absorption of low-energy electrons in metal layers [17], and so on.

Since thin or ultra-thin metal films can exhibit many intriguing properties related to fundamental physical principles, recently, the presented quantum size effects have captured the intense attention of many researchers. For example, strange metallic states, where the scaling phenomena break down because of the spontaneous formation of superconducting localized islands, emerge near the superconductor–metal quantum-phase transition in thin films [18]; size-dependent stability and oscillatory behavior (depending on the odd–even numbers of electrons) appear as the structural and electronic characteristics of charged metal clusters [19]; and novel quantum-phase transition and low-temperature electric transport effects occur in superconducting films (e.g., NbN thin epitaxial films) [20]. The quantum mechanical de Broglie matter wave of electrons can make modifications to various physical properties in thin metal films, e.g., the quantum confinement-modified heat capacity of thin films with 1∼100 nm thickness [21]; quantum-size-effect-induced Fermi-surface anisotropy; charge-carrier-surface scattering kinetics; and the resulting electric conductivity modification in thin metal films [22]. Therefore, thin or ultra-thin metal films can find a variety of potential applications, e.g., as ultra-thin metallic film-based transparent electrodes with extremely high light transmittance for photovoltaics (solar cells) and light-emitting diodes [23], exhibiting optical and electronic characteristics of metallic interfaces, and as nanoscale films at the quantum mechanical level [24,25], exhibiting symmetric or antisymmetric collective surface-plasmon modes, Landau damping, and electron–phonon scattering, which depend on the film thickness [24].

The quantum effect of size and shape dependence of films can also find some potential applications, e.g., in ultra-thin film nanoscale superconducting devices [26,27,28]. Though a number of quantum size effects have been studied for thin metal films, including thermodynamic performance (e.g., thermal stability [29] and surface energy [30,31]) and the size or shape dependence of some important thermal properties (e.g., thermal capacity) [32,33,34], less attention has been paid to the size-dependent oscillatory behavior in the electronic and lattice specific heat capacity of nanofilms. In the literature, there exists some theoretical work on the specific heat of nanostructures (e.g., spherical nanoparticles, nanotubes, or nanowires) [32,33,34], where the issues include (*i*) the dependence of specific heat on nanoscales (e.g., the radius of nanoparticles and nanotubes) when the layer numbers are fixed and (*ii*) the relationship of specific heat with temperature when the nanostructure radius is fixed. Though such specific heat behaviors can also be referred to as “quantum size effects”, the more intriguing “*even–odd layer oscillation behavior*” of specific heat capacity has not been indicated in these studies. Here, we shall study such an interesting size effect due to the quantum confinement of electron and phonon states. This quantum confinement effect (*even–odd layer oscillation behavior*) would be exhibited in both electronic and phononic specific heat.

In this paper, we shall consider the effect of the quantum confinement of both electrons and phonons, which have a strong influence on nanoscale thermal characteristics. Since the specific heat capacity—one of the most important thermal quantities—can substantially affect the growth, formation, and stability of thin metal films, we will focus on the quantum size effect of specific heat in an ultra-thin metal film and show that the specific heat resulting from both electron and phonon distribution in the metallic nanofilm depends *in oscillatory fashion* on the ultra-thin film thickness. Apart from this, the size dependence of electron Fermi wavelength in a metallic nanofilm will also be studied in this paper. We expect that the size-dependent statistical thermodynamics of nanoscale films would open a good perspective for the applications of physical and chemical properties of ultra-thin metal films and relevant low-dimensional nanomaterials.

## 2. The Electron Density of States and Quantum Size Effect of Electronic Specific Heat in an Ultra-Thin Metal Film

We shall adopt a widely used model (based on free quantum electron gas approximation) for considering the statistical properties of electrons, where a quantum gas of non-interacting electrons is confined in a potential well, in a metal. According to the present model of fermion gas, it is known in solid state physics that the electronic specific heat at low temperatures is proportional to the absolute temperature *T* and can be expressed as $C_{e}=\gamma T$, where γ is the electronic specific heat coefficient. This law of linear temperature dependence emerges because only electrons near the Fermi surface have enough thermal energy to contribute to the specific heat. However, in ultra-thin metal films, quantum confinement effects of electrons can alter the density of states near the Fermi energy level, leading to modifications in the specific heat behavior compared with bulk materials. These effects are crucial for understanding the thermal properties of nanomaterials.

In this section, we shall study the size-dependent resonance behavior of electronic specific heat that depends on the thickness of an ultra-thin metallic nanofilm. According to the statistical physics, the total internal energy *U* of electrons in a metal is given by $U=\int_{0}^{+\infty }Ef(E)N(E)dE$, where $f\left(E\right)=\frac{1}{e^{\left(E-E_{F}\right)/\left(k_{B}T\right)}+1}$ is the Fermi–Dirac distribution and N(E) is the electron density of states at the energy level *E*. The electronic specific heat capacity in a metal has been presented in Appendix A, i.e., from Equation (A1) to Equation (A8). According to the specific heat $C_{e}=\frac{\pi^{2}}{3}k_{B}N\left(E_{F}^{0}\right)\left(k_{B}T\right)$ given in Equation (A8) in Appendix A, the electronic specific heat of the metal depends on the electron density of states at Fermi energy. In a bulk metal, the electronic density of states, $N(E_{F}^{0})$, does not depend on the size and shape of the material, i.e., no quantum size or shape effects can be exhibited for a 3-D bulk material.

**Figure 1:**
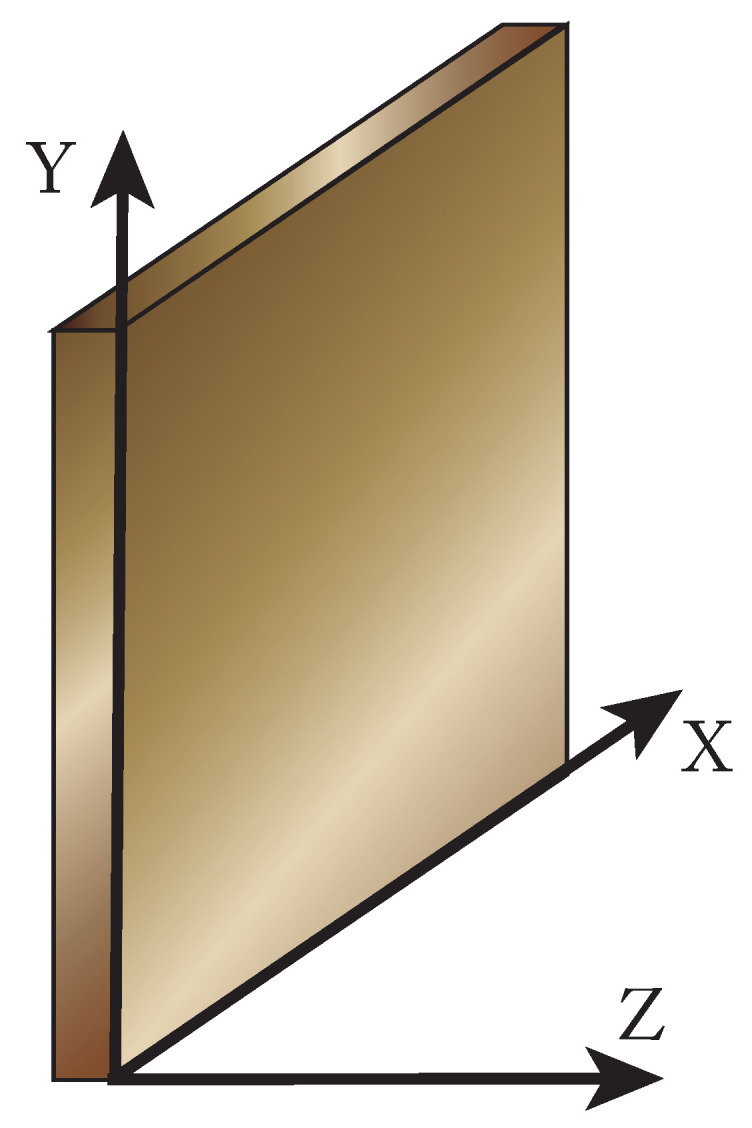
The sketch of an ultra-thin metal film. Electrons in such a thin film are unlimited in X–Y plane but quantum confined in the *Z* direction (the thickness direction) of the thin film, and there are standing wave modes of electron de Broglie matter wave in the *Z* direction. Such quantum well eigenstates in the *Z* direction are quite sensitive to the ultra-thin film thickness, because a stationary standing wave eigenstate of electrons in a quantum well requires that the thin film thickness be an integral multiple of half wavelength of the electron matter wave (in the *Z* direction). Such a requirement should also be fulfilled for phonon states caused by lattice vibration. Then, there are dramatic variations (e.g., damped oscillatory behavior) in both electronic and phononic specific heat when the thickness of ultra-thin metallic film increases.

As far as an ultra-thin metal film is concerned, the electron density of states is different from that of the bulk material due to quantum confinement of wave modes of electrons in the film thickness direction. The ultra-thin metal film with a thickness of a few atomic monolayers is schematically illustrated in Figure 1. We shall address the film electron density of states and its contribution to the electronic specific heat. In the thickness direction normal to the nanometer-sized film, the electrons form the so-called *quantum well standing wave eigenstates*, while the motion of electrons in the nanofilm plane direction can be assumed to remain free [35]. The electron density of states N(E) represents the number of electrons per unit energy and per unit volume. The total number of possible states $N_{\mathrm{tot}}$ in the ultra-thin metal film is $N_{\mathrm{tot}}=2\frac{\int dp_{x}dxdp_{y}dydp_{z}dz}{h^{3}}$, where *h* denotes the Planck constant and $p_{x},p_{y},p_{z}$ are the three momentum components of electrons. The factor 2 in $N_{\mathrm{tot}}$ is the degree of spin degeneracy of electrons. Here, $\int dp_{x}dx\int dp_{y}dy\int dp_{z}dz$ is the 6-D phase space volume. In a 2-D *x*–*y* polar coordinate plane, $\int dp_{x}dp_{y}dxdy\to L_{x}L_{y}\int 2\pi pdp$. Here, the 2-D momentum is $p=\sqrt{p_{x}^{2}+p_{y}^{2}}$ and $L_{x}=\int dx,L_{y}=\int dy$ are the lengths of the sides of the ultra-thin metal film. Then, the phase space volume in the X–Y plane is $\pi p^{2}L_{x}L_{y}$. We assume that the kinetic energy of electrons in the X–Y plane is $\varepsilon =\frac{p^{2}}{2m^{*}}$, where $m^{*}$ is the effective mass of electrons. Then, the *x*–*y* phase space volume is $\int dp_{x}dxdp_{y}dy=\int \left(2\pi m^{*}d\varepsilon \right)L_{x}L_{y}$. Since the quantum confinement effect occurs in the vertical direction (the z-axis direction), the electrons should exist in the form of quantum mechanical standing waves. Then, one can arrive at the total state number in the *Z*-direction:

(1) $$\frac{\int dp_{z}dz}{h}=\frac{\left(\int_{-p_{z}}^{p_{z}}dp_{z}\right)t}{h}=\frac{2p_{z}t}{h}=\frac{t}{\lambda /2},$$

where *t* denotes the film thickness. Since there must be an integer multiple of half wavelength of the standing waves of electrons in the vertical thickness direction (in the *z*-axis, as shown in Figure 1), the total state number $\frac{\int dp_{z}dz}{h}$ should be an integer. When $\lambda =\lambda_{F}$ (the Fermi wavelength $\lambda_{F}=\frac{h}{\sqrt{2m^{*}E_{F}}}$), the total number, $\frac{t}{\lambda /2}$, of half wavelength in the *z*-direction will be maximized. In general, however, $\frac{2t}{\lambda_{F}}$ is not an integer. Thus, one should take the form $\frac{\int dp_{z}dz}{h}=\left[\frac{2t}{\lambda_{F}}\right]$, where the symbol $\left[\frac{2t}{\lambda_{F}}\right]$ means the integer not exceeding $\frac{2t}{\lambda_{F}}$, i.e., $\left[\frac{2t}{\lambda_{F}}\right]$ is only the integer part of $\frac{2t}{\lambda_{F}}$. Now the electron density of states of the ultra-thin metal film can be written as

(2) $$N=\frac{1}{V}\frac{\partial N_{\mathrm{tot}}}{\partial \varepsilon }=2\frac{1}{V}\frac{\partial }{\partial \varepsilon }\left(\frac{\int dp_{x}dxdp_{y}dydp_{z}dz}{h^{3}}\right),$$

where $V=L_{x}L_{y}t$ is the volume of the film. So one can obtain the electron density of states of the Fermi energy level at absolute zero temperature:

(3) $$N\left(E_{F}\right)=2\frac{1}{L_{x}L_{y}t}\frac{2\pi m^{*}}{{\left(2\pi \hbar \right)}^{2}}L_{x}L_{y}\left[\frac{2t}{\lambda_{F}}\right]=\frac{m^{*}}{\pi \hbar^{2}}\frac{\left[2t/\lambda_{F}\right]}{t}.$$

This relation (3) for the density of states of free electrons at Fermi energy level in metal films is a well-known essential result for quantum size effects [12,36]. We also suggest a more rigorous formalism for deriving Equation (3), which can be found in Appendix B, i.e., Equation (A9) to Equation (A13).

Now we need to consider whether the traditional statistical thermodynamics still holds for ultra-thin metal films. Indeed, the concern about the non-continuous nature of film thickness at the sub-nanometer scale due to atomic sizes should be considered, because this highlights the discrete nature of materials at such scales. In statistical physics, the grand partition function of a fermion particle grand canonical ensemble is given by $\Xi =\prod_{i}{\left(1+e^{-\alpha -\beta \varepsilon_{i}}\right)}^{g_{i}}$ or $\ln\Xi =\sum_{i}g_{i}\ln\left(1+e^{-\alpha -\beta \varepsilon_{i}}\right)$ with the degeneracy degree $\sum_{i}g_{i}\to \frac{2}{h^{3}}\int dxdydzdp_{x}dp_{y}dp_{z}$. As is well known, in a macroscopic system consisting of a large number of particles, such as an electron gas system in a 3-D bulk solid, where the microscopic particles participate in thermal motion in a smooth, continuous 3-D spatial manifold, the 3-D coordinate space and the 3-D momentum space are independent, and hence, the degeneracy degree can be written as $\sum_{i}g_{i}\to \frac{2\pi \int dV_{\mathrm{vol}}}{h^{3}}{\left(2m\right)}^{\frac{3}{2}}\int \varepsilon^{\frac{1}{2}}d\varepsilon$, where the two integrals $\int dV_{\mathrm{vol}}$ and $\int \varepsilon^{\frac{1}{2}}d\varepsilon$ are independent.

As is known, the assumption of a continuous film thickness is an idealization often used in theoretical models to simplify calculations and obtain general trends. However, in practice, the atomic structure imposes a limit on how smoothly we can vary the thickness, particularly when we approach scales where individual atomic layers are significant. So we have to be confronted with such a question: For an ultra-thin metal film composed of only a few atomic layers, does the above linear statistically thermodynamical property (i.e., the 3-D momentum is independent of the 3-D coordinate space) still hold? The answer is yes, because what we consider is the quantum mechanical stationary states rather than scattered states, namely, we adopt the Hamiltonian eigenstate space whose energy eigenvalues $\varepsilon_{i}$ do not depend on the 3-D spatial coordinates (x,y,z). In the ultra-thin regime, especially at the sub-nanometer scale, the system of electrons can still exhibit the quantum mechanical stationary states, which correspond to the energy eigenvalues $\varepsilon_{i}$. Therefore, the integral $\int dV_{\mathrm{vol}}$ in the 3-D coordinate space and $\int \varepsilon^{\frac{1}{2}}d\varepsilon$ in the Hamiltonian eigenstate space for the partition function are independent, and the statistical thermodynamics is still valid for ultra-thin metal films.

Since we shall consider the specific heat capacity at zero temperature or a low temperature that is much lower than the film Fermi temperature, the Fermi wavelength $\lambda_{F}$ in the result (3) can be replaced by the zero-temperature Fermi wavelength $\lambda_{F}^{0}$. By substituting Equation (3) into the specific heat formula $C_{e}=\frac{\pi^{2}}{3}k_{B}N\left(E_{F}^{0}\right)\left(k_{B}T\right)$ (in solid state physics), the electronic specific heat in the ultra-thin metal film turns out to be in the form

(4) $$C_{e}^{\left(f\right)}=\frac{\pi^{2}}{3}\frac{m^{*}}{\pi \hbar^{2}}\left[\frac{2t}{\lambda_{F}^{0}}\right]\frac{\left(k_{B}T\right)k_{B}}{t}.$$

**Figure 2:**
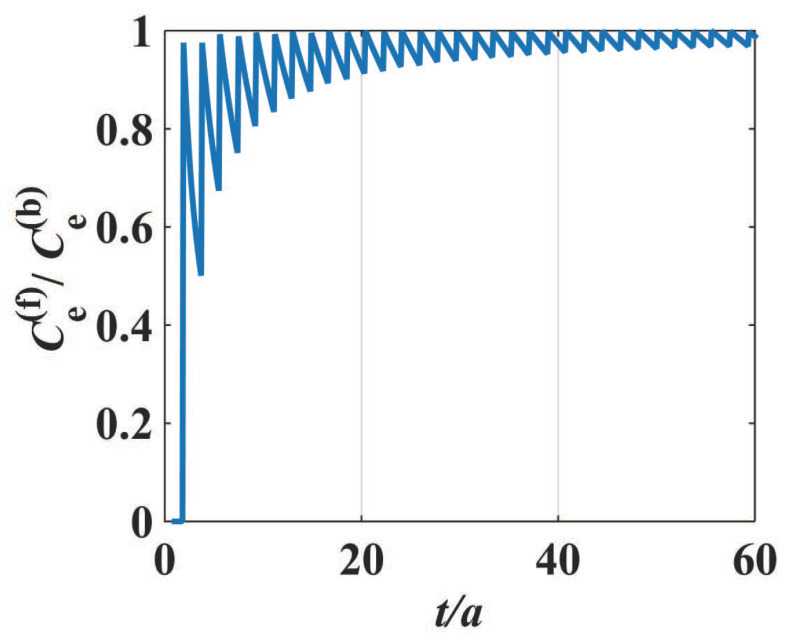
The damped oscillatory dependence of the electronic specific heat on the metal film thickness *t*. The parameter *a* is the thickness of each monolayer of atoms and t/a is the number of layers of atoms. The two quantities $C_{e}^{\left(f\right)}$ and $C_{e}^{\left(b\right)}$ denote the electronic specific heat of the 2-D *film* and 3-D *bulk* metals, respectively. When the thickness of the ultra-thin metal film is exactly an integer multiple of half wavelength of the standing waves of electrons in the thickness direction, the electron density of states would become maximized, and the electronic specific heat takes its maximum. When the thickness of the ultra-thin metal film increases, it is no longer the integer times the half wavelength, and the specific heat decreases, dropping to a minimum. If, however, the thickness becomes again a new integer multiple of the half wavelength of modes of quantum well standing wave modes of electrons, this leads to a sudden jump in the electronic density of states.

Note that there is a factor $\left[\frac{2t}{\lambda_{F}^{0}}\right]\frac{1}{t}$ in Equation (4), where $\left[\frac{2t}{\lambda_{F}^{0}}\right]$ is an integer that does not exceed $\frac{2t}{\lambda_{F}^{0}}$. If the thickness *t* of the ultra-thin metal film is exactly an integer multiple of the half wavelength of the standing waves of electrons in the vertical direction, the density of states and electronic specific heat would become maximized. However, when the thickness *t* of the ultra-thin metal film increases, $\frac{2t}{\lambda_{F}^{0}}$ is no longer the integer times the half wavelength of the electrons in the vertical direction. In this case, the integer $\left[\frac{2t}{\lambda_{F}^{0}}\right]$ remains invariant, whereas the factor $\left[\frac{2t}{\lambda_{F}^{0}}\right]\frac{1}{t}$ decreases with *t*, and finally, it falls to a minimum and encounters a next integer $\left[\frac{2t}{\lambda_{F}^{0}}\right]$. This gives rise to a sudden change in the specific heat (4), i.e., the electronic specific heat of the film jumps to a next maximum. The dependence of the electronic specific heat on the film thickness (i.e., strongly even–odd layer oscillatory behavior) is shown in Figure 2, where the film electronic specific heat $C_{e}^{\left(f\right)}$ is scaled in the unit of the 3-D bulk electronic specific heat $C_{e}^{\left(b\right)}$. It should be pointed out that such a similar oscillatory effect with the atom layer numbers in films has been derived and observed in previous references [12,36] because all such effects originate from the same density of states of free electrons at Fermi energy level in metal films.

Owing to the influence of the quantum size effect, the electron density of states of the ultra-thin metal film exhibits a characteristic of damped zigzag behavior (i.e., *stepwise decrease and sudden increase* with increasing thickness *t*), which leads to the size-dependent oscillatory behavior of the electronic specific heat when the film thickness *t* increases. It can be found that the period of oscillation is $\frac{\lambda_{F}^{0}}{2}$ and the amplitude of oscillation becomes smaller if the film thickness continues to increase. When the film thickness *t* is sufficiently large (e.g., $t≃10\lambda_{F}^{0}$), the electronic density of states given in Equation (3) will be $N\left(E_{F}^{0}\right)\to \frac{2m^{*}}{\pi \hbar^{2}\lambda_{F}^{0}}$, i.e., the electronic density of states no longer depends on the geometric size of the thin film. Under this condition of relatively large thickness, the electronic specific heat of the film reduces to that of a bulk material, i.e.,

(5) $$C_{e}^{\left(f\right)}\to \frac{2\pi k_{B}}{3}\frac{m^{*}}{\hbar^{2}}\frac{k_{B}T}{\lambda_{F}^{0}}=C_{e}^{\left(b\right)}.$$

This bulk specific heat $C_{e}^{\left(b\right)}$ is independent of the size and shape of the material, i.e., the oscillatory dependence of the electronic specific heat on the metal film thickness *t* is damped with the increasing thickness *t*, and finally, the even–odd layer oscillation in the film specific heat $C_{e}^{\left(f\right)}$ disappears, as indicated in Figure 2.

## 3. The Phonon Density of States and Lattice Specific Heat of an Ultra-Thin Metal Film

In the preceding section, we have considered the size dependence of electronic specific heat of the thin metal film. In addition to the electronic specific heat, the lattice (or phononic) specific heat also contributes to the total heat capacity of the thin metal. As is well known, the lattice specific heat origins from lattice vibration, and it depends on the density of states of phonons. It can be found that such phononic specific heat of a nanofilm also shows significant size dependence due to quantum confinement of phonons (in the thickness direction of the ultra-thin film). The phonon dispersion relation is ℏω=ℏkc, with *c* being the acoustic speed. However, one needs to replace *c* with the effective acoustic speed $\bar{c}$, which satisfies $\frac{1}{{\bar{c}}^{3}}=\frac{1}{3}\left(\frac{1}{c_{l}^{3}}+\frac{2}{c_{t}^{3}}\right)$, since each wave vector k of phonons corresponds to one longitudinal wave mode and two independent transverse wave modes [37,38,39]. Here, $c_{l}$ and $c_{t}$ denote the acoustic wave speeds of longitudinal and transverse modes, respectively.

We shall use the Debye model for studying the thermal characteristics of the ultra-thin metal films. The Debye model is a widely used approach in describing the lattice vibrations (phonon modes) and the contribution to specific heat for a solid, particularly at low temperatures [37,38,39]. It supposes that the phonon spectrum can be described or approximated by a frequency-continuous distribution of lattice vibrational modes up to a maximum phonon frequency (i.e., the so-called Debye frequency), where the specific heat capacity is calculated by integrating over all these lattice vibrational modes. At low temperatures, the Debye model of a solid material predicts a $T^{3}$-law dependence of the lattice/phonon specific heat capacity, which gradually tends to a constant value (i.e., the Dulong–Petit law) at higher temperatures. This model is especially important for understanding the temperature dependence of specific heat in ultra-thin metal films, as quantum confinement effects can modify the phonon spectrum, influencing the specific heat behavior.

Now we shall calculate the density of states of phonons in a thin film. The degree of freedom of atoms in the present metal film is

(6) $$\begin{array}{ccc} 3N_{\mathrm{atom}} & = & \sum_{k}\to \sum_{k_{z}}\frac{3}{{\left(2\pi \right)}^{2}}\int_{S}dxdy\int_{k_{⊥}}dk_{x}dk_{y}\\ & = & \sum_{k_{z}}\frac{3S}{{\left(2\pi \right)}^{2}}\int_{0}^{k_{⊥}}2\pi k_{xy}dk_{xy}\\ & = & \sum_{n=1}^{n_{\max}}\frac{3S}{{\left(2\pi \right)}^{2}}\pi \left[k_{D}^{2}-{\left(\frac{n\pi }{t}\right)}^{2}\right], \end{array}$$

where $N_{\mathrm{atom}}$ denotes the total number of atoms in the metal film and $k_{x},k_{y}$ are the wave numbers of phonon (lattice) modes in the *x*–*y* plane of the thin film. The standing mode wave number of phonons is $k_{z}=\frac{n\pi }{t}$ (in the thickness direction). The film area is $S=\int_{S}dxdy$, and the transverse wave vector space $\int_{k_{⊥}}dk_{x}dk_{y}=\pi k_{xy}^{2}{|}_{0}^{k_{⊥}}$ with $k_{xy}=\sqrt{k_{x}^{2}+k_{y}^{2}}$. Note that $k_{⊥}$ is the allowed maximum of $k_{xy}$ in the *x*–*y* plane. Clearly, the maximum value of $k_{xy}^{2}$ is $k_{⊥}^{2}=k_{D}^{2}-k_{z}^{2}=k_{D}^{2}-{\left(\frac{n\pi }{t}\right)}^{2}$, where $k_{D}$ is the Debye wave number (corresponding to the Debye frequency $\omega_{D}=k_{D}\bar{c}$). Since $k_{z}=\frac{n\pi }{t}$, the allowed maximum of the integer *n* of the standing mode wave number $k_{z}$ of phonons in the relation (6) is $n_{\max}<\frac{k_{D}t}{\pi }$, namely, $n_{\max}$ is the integer part of $\frac{k_{D}t}{\pi }$, i.e., $n_{\max}=\left[\frac{k_{D}t}{\pi }\right]$. The relation (6) can be used to obtain the Debye wave number $k_{D}$ (and hence the Debye frequency $\omega_{D}$) of lattice vibration in an ultra-thin film. Obviously, it can be found from the relation (6) that the Debye wave number $k_{D}$ is no longer a constant, i.e., it depends on the film thickness *t* when the film has only a few atom layers.

Since the degree of freedom of atoms equals the total mode number, *M*, of lattice vibration (including transverse and longitudinal waves), the total mode number, of which the frequencies are smaller than ω (here, $\omega \leq \omega_{D}$), is $M\left(\omega \right)=\sum_{k_{z}}\frac{3S}{{\left(2\pi \right)}^{2}}\pi k_{⊥}^{2}=\sum_{k_{z}}\frac{3S}{{\left(2\pi \right)}^{2}{\bar{c}}^{2}}\pi \omega_{⊥}^{2}$ with $\omega_{⊥}^{2}=k_{⊥}^{2}{\bar{c}}^{2}$. Since $\omega^{2}=\omega_{⊥}^{2}+{\left(\frac{n\pi }{t}\right)}^{2}{\bar{c}}^{2}$, the mode number of phonons turns out to be in the form

(7) $$M\left(\omega \right)=\sum_{n=1}^{n_{\max}}\frac{3S}{{\left(2\pi \right)}^{2}{\bar{c}}^{2}}\pi \left[\omega^{2}-{\left(\frac{n\pi }{t}\right)}^{2}{\bar{c}}^{2}\right].$$

Clearly, the integer *n* is less than $\frac{\omega t}{\pi \bar{c}}$, and the maximum integer $n_{\max}$ is the integer part of $\frac{\omega t}{\pi \bar{c}}$, i.e., $n_{\max}=\left[\frac{\omega t}{\pi \bar{c}}\right]$, where ω is less than the allowed maximum frequency (the Debye frequency $\omega_{D}$) of lattices. Thus, the density of states of phonons at the frequency ω is given by

(8) $$g\left(\omega \right)=\frac{1}{V}\frac{\delta M(\omega )}{\delta \omega }=\sum_{n=1}^{n_{\max}}\frac{6}{{\left(2\pi \right)}^{2}{\bar{c}}^{2}}\frac{\pi }{t}\omega =\frac{6}{{\left(2\pi \right)}^{2}{\bar{c}}^{2}}\frac{\pi }{t}\omega n_{\max}.$$

Then the phonon density of states of the ultra-thin metal film (with the thickness at nanoscale) can be written as

(9) $$g\left(\omega \right)=\frac{6}{{\left(2\pi \right)}^{2}{\bar{c}}^{2}}\frac{\pi }{t}\omega \left[\frac{\omega t}{\pi \bar{c}}\right].$$

It should be pointed out that the specific heats of fermions and bosons are different because of their respective quantum statistical characteristics (the distinct Fermi–Dirac and Bose–Einstein statistics). For example, only the electrons at the Fermi surface make the contribution to the electronic specific heat, as suggested by Equation (A8). Thus, the size dependence of electronic specific heat is determined by the density of states of electrons at Fermi level. For the phonons, however, all the modes with frequencies less than the Debye frequency $\omega_{D}$ will contribute to the phononic specific heat. Therefore, the phononic specific heat depends on the density of states of phonons with all the mode frequencies below the Debye frequency $\omega_{D}$. According to the Debye model of solid lattices and the effect of lattice quantization [37,38,39], the lattice specific heat of a material can take the form

(10) $$C_{p}^{\left(f\right)}=k_{B}\int_{0}^{\omega_{D}}\frac{{\left(\frac{\hbar \omega }{k_{B}T}\right)}^{2}\exp\left(\frac{\hbar \omega }{k_{B}T}\right)}{{\left[\exp\left(\frac{\hbar \omega }{k_{B}T}\right)-1\right]}^{2}}g\left(\omega \right)d\omega .$$

Substitution of the density of states (9) into Equation (10) yields the lattice specific heat of the ultra-thin metal film

(11) $$C_{p}^{\left(f\right)}=\frac{3k_{B}}{2\pi {\bar{c}}^{2}t}\int_{0}^{\omega_{D}}\frac{{\left(\frac{\hbar \omega }{k_{B}T}\right)}^{2}\exp\left(\frac{\hbar \omega }{k_{B}T}\right)}{{\left[\exp\left(\frac{\hbar \omega }{k_{B}T}\right)-1\right]}^{2}}\omega \left[\frac{\omega t}{\pi \bar{c}}\right]d\omega .$$

**Figure 3:**
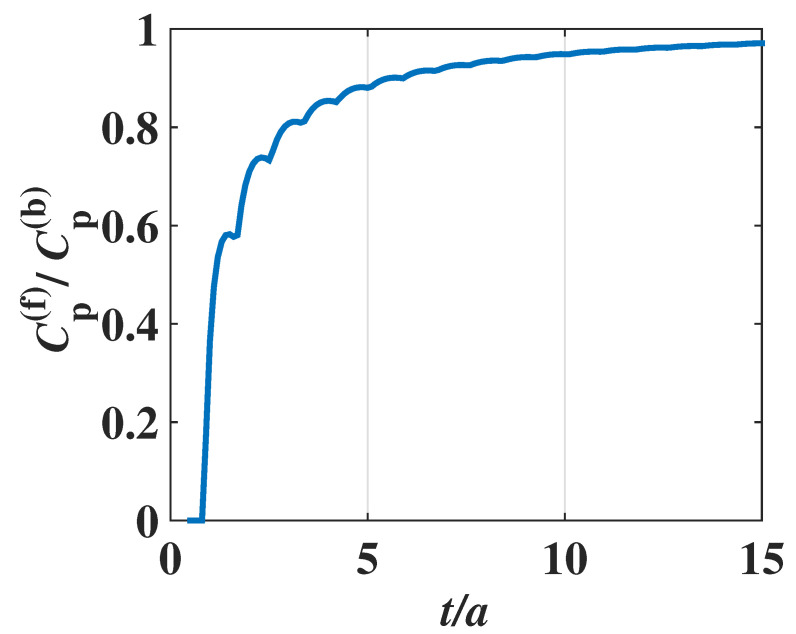
The weakly even–odd layer oscillatory behavior (i.e., damped oscillatory dependence on the film thickness *t*) of the phononic specific heat. The parameter *a* is the thickness of a single layer and t/a is the number of layers of atoms. The two quantities $C_{p}^{\left(f\right)}$ and $C_{p}^{\left(b\right)}$ denote the phononic specific heat of the 2-D film and 3-D bulk materials, respectively. The characteristics of size dependence of the phononic specific heat of the ultra-thin film include the following. (i) The film phononic specific heat $C_{p}^{\left(f\right)}$ increases with the thickness *t* (global behavior) and (ii) $C_{p}^{\left(f\right)}$ exhibits small fluctuation with damped amplitude (local behavior).

**Figure 4:**
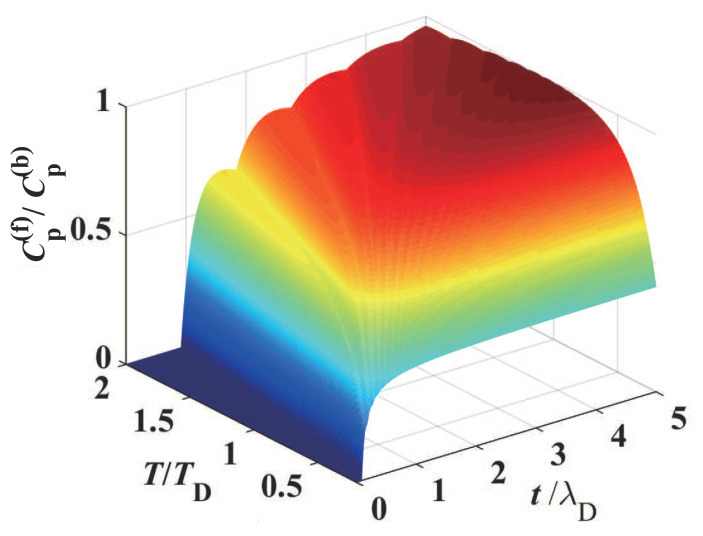
The phononic specific heat of film depending on the thickness *t* and the temperature *T*. When the metal film thickness *t* is small, the specific heat of the film is also relatively low (represented by the blue region in the figure), whereas when the film thickness *t* is large, the specific heat of the film is relatively high (represented by the red region in the figure). The parameter $\lambda_{D}$ is the phononic Debye wavelength and $T_{D}$ denotes the Debye temperature ($T_{D}=\frac{\hbar \omega_{D}}{k_{B}}$). Though the phononic specific heat increases with the film thickness *t*, there are some small fluctuations with damped amplitudes. When the thickness *t* is large, the phononic specific heat of the *film* will finally tend to that of the *bulk* material, i.e., $C_{p}^{\left(f\right)}\to C_{p}^{\left(b\right)}$.

Now we will compare the film lattice specific heat with that of the bulk material. The dependence of lattice specific heat on the film thickness *t* (i.e., weakly even–odd layer oscillatory behavior) is shown in Figure 3 and Figure 4. There is the small thickness-dependent oscillatory (or fluctuation) behavior of the lattice specific heat. The fluctuation amplitude in the specific heat decreases when the film thickness *t* increases, and finally, the film specific heat of the phonons tends to a smooth one, i.e., the thickness-independent specific heat of a bulk material, if the film thickness *t* is sufficiently large. It can be found that the lattice specific heat (11) of the metal film can truly reduce to that of the 3-D *bulk* metal:

(12) $$C_{p}^{\left(b\right)}=\frac{3k_{B}}{2\pi^{2}{\bar{c}}^{3}}\int_{0}^{\omega_{D}}\frac{{\left(\frac{\hbar \omega }{k_{B}T}\right)}^{2}\exp\left(\frac{\hbar \omega }{k_{B}T}\right)}{{\left[\exp\left(\frac{\hbar \omega }{k_{B}T}\right)-1\right]}^{2}}\omega^{2}d\omega .$$

This lattice specific heat is independent of the thickness size.

The ratio of the lattice specific heat of *film* and *bulk* metals is given by

(13) $$\frac{C_{p}^{\left(f\right)}}{C_{p}^{\left(b\right)}}=\frac{\pi \bar{c}}{t}\cdot \frac{\int_{0}^{\omega_{D}}\frac{{\left(\frac{\hbar \omega }{k_{B}T}\right)}^{2}\exp\left(\frac{\hbar \omega }{k_{B}T}\right)}{{\left[\exp\left(\frac{\hbar \omega }{k_{B}T}\right)-1\right]}^{2}}\omega \left[\frac{\omega t}{\pi \bar{c}}\right]d\omega }{\int_{0}^{\omega_{D}}\frac{{\left(\frac{\hbar \omega }{k_{B}T}\right)}^{2}\exp\left(\frac{\hbar \omega }{k_{B}T}\right)}{{\left[\exp\left(\frac{\hbar \omega }{k_{B}T}\right)-1\right]}^{2}}\omega^{2}d\omega }.$$

It can be found from Figure 3 that the behavior of phononic specific heat depending on the film thickness *t* is different from that of the electronic specific heat. In the case of electronic specific heat, the density of states of electrons at Fermi level would be maximized, and the electronic specific heat becomes a maximum when the thickness *t* is exactly an integer multiple of half wavelength of the standing waves in the thickness direction. Then, the specific heat decreases drastically with thickness, dropping to a minimum, as is shown in Figure 2. Because of different statistics for electrons and phonons, such a characteristic of *stepwise decrease and sudden increase* exhibited in the electronic specific heat (shown in Figure 2) no longer appears in the phononic specific heat (shown in Figure 3). Instead, the amplitude of fluctuation in the phononic specific heat depending on the film thickness is quite small, and such a small fluctuation is damped as the thickness *t* increases. When the thickness is sufficiently large, the ripples on the specific heat curve disappear and the phononic specific heat of the film approaches that of the 3-D bulk material.

It should be pointed out that the curve in Figure 3, which indicates the phononic specific heat of film, is similar to that in Figure 2 of metal film electron number density in Ref. [36], but their physical meanings are completely different. The reason for interpreting why they have the similarity in the curves lies in the fact that the mode number of phonons in Equation (7) has a mathematical form similar to the total free electron number at zero temperature, which will be given in Equation (17), which is consistent with the result (10) in Ref. [36].

In the above, we have assumed that the Debye frequency $\omega_{D}$ of the phonon spectra is a constant for a given metal film (i.e., independent of the size of the thin film). But in fact, the Debye frequency $\omega_{D}$ of phonons can also depend on the ultra-thin film thickness *t*. Now we shall consider this issue:

In the statistical physics of phonon spectra in solid state physics, the relation between the Debye frequency $\omega_{D}$ and the solid atomic numbers $N_{\mathrm{atom}}$ is $3N_{\mathrm{atom}}=\int_{0}^{\omega_{D}}\frac{\delta M(\omega )}{\delta \omega }d\omega \to V\int_{0}^{\omega_{D}}g\left(\omega \right)d\omega$, with *V* being the volume. By using the phonon density g(ω) of states in the ultra-thin metal film given in Equation (9), we can obtain the following relation:

(14) $$\begin{array}{ccc} & & \int_{0}^{\omega_{D}}g\left(\omega \right)d\omega =\frac{3N_{\mathrm{atom}}}{V}\\ & \Rightarrow & \int_{0}^{\omega_{D}}\frac{6}{{\left(2\pi \right)}^{2}{\bar{c}}^{2}}\frac{\pi }{t}\omega \left[\frac{\omega t}{\pi \bar{c}}\right]d\omega =\frac{3N_{\mathrm{atom}}}{V}, \end{array}$$

where ω is less than the allowed maximum frequency (the Debye frequency $\omega_{D}$).

The result in Equation (14) can be used to determine the Debye frequency (corresponding to the thickness *t*) of an ultra-thin metal film. The quantity $\frac{1}{t}\left[\frac{\omega t}{\pi \bar{c}}\right]$, where $\left[\frac{\omega t}{\pi \bar{c}}\right]$ is the integer part of $\frac{\omega t}{\pi \bar{c}}$, can grow with jagged ripples in its curve as the thickness *t* increases. But finally, it approaches a constant $\frac{\omega }{\pi \bar{c}}$ (independent of the thickness *t*) when *t* is sufficiently large.

For a 3-D bulk metal material (corresponding to the film thickness t→∞), the above Equation (14) can be rearranged as $g\left(\omega \right)\to \frac{6}{{\left(2\pi \right)}^{2}{\bar{c}}^{3}}\omega^{2}$ and $\int_{0}^{\omega_{D}}\frac{6}{{\left(2\pi \right)}^{2}{\bar{c}}^{3}}\omega^{2}d\omega =\frac{3N_{\mathrm{atom}}}{V}$. Then, the bulk Debye frequency $\omega_{D}$ can be obtained through the result $\frac{1}{2\pi^{2}}\frac{\omega_{D}^{3}}{{\bar{c}}^{3}}=\frac{3N_{\mathrm{atom}}}{V}$.

## 4. Quantum Size Modification to the Fermi Wave Number of Electrons in a Thin Metal Film

It should be pointed out that in the preceding sections, both the electron Fermi wavelength $\lambda_{F}$ and the phonon Debye frequency $\omega_{D}$ of the thin film are identified as constant numbers, namely, the thickness dependence of the Fermi wavelength $\lambda_{F}$ of electrons and the Debye frequency $\omega_{D}$ of lattices have not been taken into account. As a matter of fact, such variations in $\lambda_{F}$ and $\omega_{D}$ would be significant for nanofilms with thickness of only a few atomic monolayers (e.g., the layer number is less than ten). As far as the ultra-thin film with more than ten atomic monolayers, the size dependence of both the Fermi wavelength of electrons and the Debye frequency of lattices can be ignored. Thus, the results given in the preceding sections are valid only for the ultra-thin metal films with sufficiently large number of atomic monolayers. In other words, for the ultra-thin films with small numbers of atomic monolayers, the quantum size modification to the Fermi wavelength of free electrons and to the Debye wavelength of phonons should be taken into account. In what follows, we will discuss the issue of quantum size correction to the electron Fermi wavelength.

### 4.1. Quantum Size Correction to the Electron Fermi Wavelength in Ultra-Thin Metal Films

In a 3-D bulk metal, at the zero temperature or low temperature (i.e., $T\ll T_{F}$), the total number of free electrons (derived within the framework of statistical physics) can be expressed as

$$N_{\mathrm{tot}}=g_{s}\sum_{k}\to g_{s}\frac{1}{{\left(2\pi \right)}^{3}}\int dk_{x}dxdk_{y}dydk_{z}dz,$$

where $dk_{x}dxdk_{y}dydk_{z}dz$ is the 6-D phase space volume element and *g* denotes the degree of spin degeneracy of electrons. Since an electron has spin-up and spin-down states, the degree of degeneracy is $g_{s}=2$. In an isotropic phase space, the above equation can be rearranged as $N_{\mathrm{tot}}=\frac{g_{s}V}{{\left(2\pi \right)}^{3}}\frac{4\pi }{3}{\left(k_{F}^{\left(3D\right)}\right)}^{3}$. Define the number density of electrons $\rho_{e}=\frac{N_{\mathrm{tot}}}{V}$, and we will have

(15) $$\begin{array}{c} \rho_{e}=\frac{g_{s}}{{\left(2\pi \right)}^{3}}\frac{4\pi }{3}{\left(k_{F}^{\left(3D\right)}\right)}^{3}, \end{array}$$

where V=∫dxdydz is the 3-D ordinary space volume. From the result (15), one can obtain the Fermi wave number of electrons in the 3-D bulk metal. The result is given by $k_{F}^{\left(3D\right)}=2\pi {\left(\frac{3\rho_{e}}{4\pi g_{s}}\right)}^{1/3}$. Such a Fermi wave number is independent of the size of the bulk material.

Now let us consider the Fermi wave number in an ultra-thin metallic film. If the metallic film thickness *t* is less than the electron Fermi wavelength $\lambda_{F}^{\left(3D\right)}$, or *t* has the same order of magnitude as $\lambda_{F}^{\left(3D\right)}$ (e.g., $\lambda_{F}^{\left(3D\right)}=1.06$ nm for metal Pb [12]), in the case of this ultra-thin film, the Fermi wavelength would be dramatically modified by the thickness *t*. Only when the film thickness *t* is much larger than the electron Fermi wavelength can the 2-D film Fermi wavelength tend to the 3-D bulk Fermi wavelength $\lambda_{F}^{\left(3D\right)}$. The total electron number in the thin film is given by

(16) $$\begin{array}{ccc} N_{\mathrm{tot}} & = & g_{s}\sum_{k}\to g_{s}\sum_{k_{z}}\frac{1}{{\left(2\pi \right)}^{2}}\int_{S}dxdy\int_{k_{⊥}}dk_{x}dk_{y}\\ & = & g_{s}\sum_{n}\frac{S}{{\left(2\pi \right)}^{2}}\pi \left[k_{F}^{2}-{\left(\frac{n\pi }{t}\right)}^{2}\right], \end{array}$$

where $S=\int_{S}dxdy$ is the area of film in the *x*–*y* plane (see the schematic diagram Figure 1) and $k_{⊥}$ is the transverse wave number of electrons in the *x*–*y* plane (film). In the $k_{x}$–$k_{y}$ wave vector space, $\int_{k_{⊥}}dk_{x}dk_{y}=\int 2\pi k_{⊥}dk_{⊥}$ with $k_{⊥}=\sqrt{k_{x}^{2}+k_{y}^{2}}$. The square of $k_{⊥}$ is given by $k_{⊥}^{2}=k_{F}^{2}-k_{z}^{2}=k_{F}^{2}-{\left(\frac{n\pi }{t}\right)}^{2}$. Since the discrete wave number of free electrons in the *z*-direction is $k_{z}=\frac{n\pi }{t}$, where $n=1,2,...,n_{\max}$, the total electron number in the thin film appears to be of the form

(17) $$\begin{array}{ccc} N_{\mathrm{tot}} & = & g_{s}\sum_{n=1}^{n_{\max}}\frac{S}{{\left(2\pi \right)}^{2}}\pi \left[k_{F}^{2}-{\left(\frac{n\pi }{t}\right)}^{2}\right]\\ & = & \frac{g_{s}S}{{\left(2\pi \right)}^{2}}\pi \left[n_{\max}k_{F}^{2}-\frac{n_{\max}\left(n_{\max}+1\right)\left(2n_{\max}+1\right)}{6}{\left(\frac{\pi }{t}\right)}^{2}\right], \end{array}$$

where we have used the number progression sum formula $\sum_{n=1}^{n_{\max}}n^{2}=1^{2}+2^{2}+...+n_{\max}^{2}=\frac{n_{\max}\left(n_{\max}+1\right)\left(2n_{\max}+1\right)}{6}$. Since the electron wave number in the thickness direction is $k_{z}=\frac{n\pi }{t}$ and the maximum ${\left(k_{z}\right)}_{\max}=\frac{n_{\max}\pi }{t}\leq k_{F}$, the allowed maximum integer is $n_{\max}=\left[\frac{k_{F}t}{\pi }\right]$. Here, the symbol $\left[\frac{k_{F}t}{\pi }\right]$ means the integer not exceeding $\frac{k_{F}t}{\pi }$, i.e., $\left[\frac{k_{F}t}{\pi }\right]$ is only the integer part of $\frac{k_{F}t}{\pi }$.

**Figure 5:**
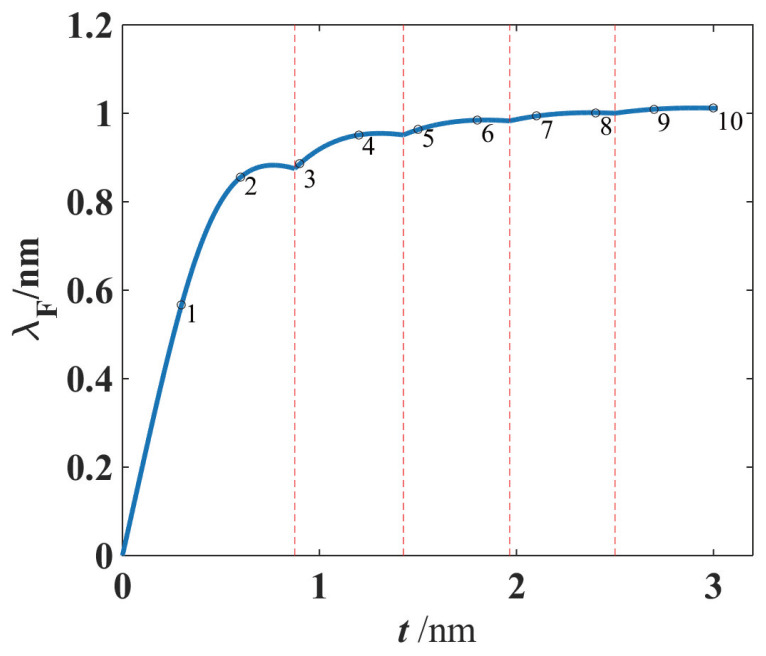
The film Fermi wavelength $\lambda_{F}$ of electrons depending on the thickness of the ultra-thin film. Because of electron standing wave mode structure in the vertical direction of the metal film, the Fermi wavelength increases with small damped fluctuation amplitude when the film thickness *t* increases. The integers 1,2,3,… on the curve represent the layer numbers of the film. When the film thickness *t* is adequately large, the film Fermi wavelength will approach that of a bulk material, i.e., finally, it no longer changes with the increasing thickness *t*.

As an illustrative example, we will address the ultra-thin film Fermi wavelength. If the 3-D bulk Fermi wavelength $\lambda_{F}^{\left(3D\right)}=1.06$ nm and the lattice constant a=0.30 nm, it can be found by using Equation (17) that the allowed solutions of the ultra-thin film Fermi wavelength $\lambda_{F}^{\left(\mathrm{film}\right)}=\frac{2\pi }{k_{F}}$ are as follows: (i) $N_{\mathrm{layer}}=1$, $n_{\max}=1$, $\lambda_{F}^{\left(\mathrm{film}\right)}=0.57\;\mathrm{nm}$, (ii) $N_{\mathrm{layer}}=2$, $n_{\max}=1$, $\lambda_{F}^{\left(\mathrm{film}\right)}=0.86\;\mathrm{nm}$, (iii) $N_{\mathrm{layer}}=3$, $n_{\max}=2$, $\lambda_{F}^{\left(\mathrm{film}\right)}=0.89\;\mathrm{nm}$, and (iv) $N_{\mathrm{layer}}=4$, $n_{\max}=2$, $\lambda_{F}^{\left(\mathrm{film}\right)}=0.95\;\mathrm{nm}$. The film Fermi wavelength $\lambda_{F}$ of the free electrons depending on the thickness in the ultra-thin film is plotted in Figure 5. It can be seen that when the atomic monolayer number $N_{\mathrm{layer}}$ of the ultra-thin film is small, the film Fermi wavelength $\lambda_{F}^{\left(\mathrm{film}\right)}$ varies dramatically as $N_{\mathrm{layer}}$ changes. If, however, the film thickness *t* is sufficiently large, the result (17) reduces to the form

(18) $$\begin{array}{ccc} N_{\mathrm{tot}} & \to & \frac{g_{s}S}{{\left(2\pi \right)}^{2}}\pi \left[n_{\max}k_{F}^{2}-\frac{n_{\max}^{3}}{3}{\left(\frac{\pi }{t}\right)}^{2}\right]\\ & \to & \frac{g_{s}S}{{\left(2\pi \right)}^{2}}\pi \left[\frac{k_{F}^{3}t}{\pi }-\frac{{\left(\frac{k_{F}t}{\pi }\right)}^{3}}{3}{\left(\frac{\pi }{t}\right)}^{2}\right]\\ & = & \frac{g_{s}S}{{\left(2\pi \right)}^{2}}\pi \frac{2k_{F}^{3}t}{3\pi }, \end{array}$$

where, since $n_{\max}=\left[\frac{k_{F}t}{\pi }\right]$, the allowed maximum integer of Fermi surface electron half wavelength in the thickness direction is $n_{\max}≃\frac{k_{F}t}{\pi }$. Thus, the number density (the number of electrons per unit volume) is $\rho_{e}=\frac{N_{\mathrm{tot}}}{St}=\frac{g_{s}}{{\left(2\pi \right)}^{3}}\frac{4\pi }{3}k_{F}^{3}$, which is consistent with the result (15) of the 3-D bulk metallic material.

The free electron number density $\frac{N_{\mathrm{tot}}}{St}$ (for the metal film) obtained from the above result (17) is consistent with the result in Ref. [36], where the author plotted the curve of free electron number density $n_{e}$ versus $\frac{k_{F}t}{\pi }$. But in our understanding, for a given metal, whether the metal is a bulk material or a film, the free electron number density $n_{e}$ is almost a fixed constant number whose magnitude is proportional to the atomic number density. Then, we believe that it is the free electron number density that determines the Fermi wave number $k_{F}$, that is, in this physical mechanism, the free electron number density is a cause and the Fermi wave number is an effect. For metal films, electron number density $n_{e}$ and thickness *t* together determine the Fermi wave number $k_{F}$, so the aforementioned result such as Equation (17) and Formula (10) in Dong’s paper [36] should be understood as a relation between the electron Fermi wave number $k_{F}$ and the changes in $n_{e}$ and *t*. In Ref. [36], it may appear that the Fermi wave number was treated as a fixed value (independent of thickness), so Figure 2 in Ref. [36] shows that the free electron number density is determined by the thickness *t* of the metal film. But we prefer to believe that for a given metal film, its free electron number density is a fixed constant (not obviously dependent on the thickness of the metal film), so Formula (17) in our paper reflects the Fermi wave number or Fermi wavelength that is determined by the metal thickness, which is shown in our Figure 5. In a word, although the above Formula (17) is mathematically equivalent to Dong’s Formula (10) [36], our interpretation of the physical meanings of Equation (17) would be completely different from Dong’s.

As has been pointed out in this section, for the films with more than ten atomic layers, the quantum size corrections to the Fermi wavelength and the Debye frequency are small and can almost be ignored. For this reason, the electron Fermi wavelength $\lambda_{F}$ and the phonon Debye frequency $\omega_{D}$ can be identified as constant numbers (i.e., they do not change much when the ultra-thin film thickness *t* increases). However, there still exists distinct oscillatory behaviors of both electronic and phononic specific heat in this situation.

In our simulation shown in the previous sections, the 3-D bulk Fermi wavelength $\lambda_{F}$, Debye wavelength $\omega_{D}$, and the lattice constant *a* are chosen as 1.06 nm, 2.50 nm, and 0.286 nm, respectively, for the metal, e.g., Pb, and the dynamical responses of the specific heat depending on the atomic layer numbers have been theoretically demonstrated. It can be found that the oscillatory behaviors are still considerable for the films with less than 50 atomic layers, as shown in Figure 2 and Figure 3, where the x-axes are scaled in the unit of atomic layers (i.e., the layer number is t/a). When the film thickness is sufficiently large, e.g., more than 50 atomic layers, the fluctuation behavior gradually becomes weak, and so the total specific heat reduces to that of a 3-D bulk material.

### 4.2. Comparison of the Two Cases with and without Quantum Size Correction to the Fermi Wavelength

In the research of quantum size effects for many phenomena in lower-dimensional materials, in general, the size correction to the electron Fermi wavelength was often not taken into account. Since the electron mode distribution in the momentum space depends on the film size, we shall study and calculate the electron densities of states (at the Fermi energy level) that are modified by the “Fermi wavelength correction”.

According to Equation (3), the density of states of electrons at the Fermi surface of a thin metal film is given by $N^{\left(f\right)}\left(E_{F}\right)=\frac{m^{*}}{\pi \hbar^{2}}\frac{\left[\frac{2t}{\lambda_{F}^{\left(c\right)}}\right]}{t}$, where $\left[\frac{2t}{\lambda_{F}^{\left(c\right)}}\right]$ denotes the number of half wavelength of electron. When the film thickness t→∞ (or sufficiently large), the density $N^{\left(f\right)}\left(E_{F}\right)$ of states of electrons in the *film* approaches that of a *bulk* material, i.e.,

(19) $$\begin{array}{c} N^{\left(f\right)}\left(E_{F}\right)\to \lim_{t\to \infty }\frac{m^{*}}{\pi \hbar^{2}}\frac{\left[\frac{2t}{\lambda_{F}^{\left(c\right)}}\right]}{t}=\frac{m^{*}}{\pi \hbar^{2}}\frac{2}{\lambda_{F}^{\left(c\right)}}\equiv N^{\left(b\right)}\left(E_{F}\right), \end{array}$$

where $N^{\left(b\right)}\left(E_{F}\right)$ denotes the electron density of states at the Fermi level in the *bulk* metal. Therefore, the ratio of state density $N^{\left(f\right)}\left(E_{F}\right)$ (for a film) to $N^{\left(b\right)}\left(E_{F}\right)$ (for a bulk) is given by

(20) $$\begin{array}{c} R_{1}=\frac{N^{\left(f\right)}\left(E_{F}\right)}{N^{\left(b\right)}\left(E_{F}\right)}=\frac{\frac{m^{*}}{\pi \hbar^{2}}\frac{\left[\frac{2t}{\lambda_{F}^{\left(c\right)}}\right]}{t}}{\frac{m^{*}}{\pi \hbar^{2}}\frac{2}{\lambda_{F}^{\left(c\right)}}}=\frac{\lambda_{F}^{\left(c\right)}}{2t}\left[\frac{2t}{\lambda_{F}^{\left(c\right)}}\right]. \end{array}$$

Since $\left[\frac{2t}{\lambda_{F}^{\left(c\right)}}\right]$ represents the integer part of $\frac{2t}{\lambda_{F}^{\left(c\right)}}$, this means the ratio $R_{1}\leq 1$. When the thickness *t* is sufficiently large, $R_{1}\to 1$ (i.e., the electron density of states of the metal film approaches that of the bulk metal).

It should be pointed out that in the above theoretical model, the behavior of dependence of the electron Fermi wavelength on the film thickness is ignored, i.e., for the thin film, we have chosen a constant Fermi wavelength $\lambda_{F}^{\left(c\right)}$ that is independent of the film thickness *t*, as has been shown in Equations (19) and (20). Such a constant $\lambda_{F}^{\left(c\right)}$ can be adopted as the Fermi wavelength in the bulk metal material.

As a matter of fact, however, in an ultra-thin metal film, the Fermi wavelength must unavoidably depend on the film thickness *t* or on the atom layer number $N_{\mathrm{layer}}$, i.e., $\lambda_{F}=\lambda_{F}\left(N_{\mathrm{layer}}\right)$. We need to revisit the ratio of free electron state density $N^{\left(f\right)}\left(E_{F}\right)$ (for a film) to $N^{\left(b\right)}\left(E_{F}\right)$ (for a bulk). The result is given as follows:

(21) $$\begin{array}{c} \begin{array}{cc} N^{\left(f\right)}\left(E_{F}\right) & =\frac{m^{*}}{\pi \hbar^{2}}\frac{\left[\frac{2t}{\lambda_{F}\left(N_{\mathrm{layer}}\right)}\right]}{t}=\frac{m^{*}}{\pi \hbar^{2}}\frac{\left[\frac{2N_{\mathrm{layer}}a}{\lambda_{F}\left(N_{\mathrm{layer}}\right)}\right]}{N_{\mathrm{layer}}a},\\ N^{\left(b\right)}\left(E_{F}\right) & =\frac{m^{*}}{\pi \hbar^{2}}\frac{2}{\lambda_{F}^{\left(c\right)}}, \end{array} \end{array}$$

where the film thickness is $t=N_{\mathrm{layer}}a$, with being *a* the thickness of a single layer of atoms. Though the relation between the lattice constant and the atomic radius in metal is influenced by the spatial periodic arrangement structure of atoms, there is a proportional correlation between the lattice constant and the atomic radius because the atoms in the metal lattices are closely arranged, and hence, the thickness of each layer of atoms in a metal film can be regarded as the magnitude of the diameter of metal atoms. Then, from the result in Equation (21), the ratio of free electron state density $N^{\left(f\right)}\left(E_{F}\right)$ (for a film) to $N^{\left(b\right)}\left(E_{F}\right)$ (for a bulk) is

(22) $$\begin{array}{c} R_{2}=\frac{N^{\left(f\right)}\left(E_{F}\right)}{N^{\left(b\right)}\left(E_{F}\right)}=\frac{\lambda_{F}^{\left(c\right)}}{2N_{\mathrm{layer}}a}\left[\frac{2N_{\mathrm{layer}}a}{\lambda_{F}\left(N_{\mathrm{layer}}\right)}\right]. \end{array}$$

Different from the result in Equation (20), which is independent of the thin film thickness ($t=N_{\mathrm{layer}}a$), the present result (22) depends on the thin film thickness.

In the above, we have shown that the ratios of the electron Fermi surface density of states of ultra-thin metal film to bulk metal [i.e., $\frac{N^{\left(f\right)}\left(E_{F}\right)}{N^{\left(b\right)}\left(E_{F}\right)}$ in Equations (20) and (22)] are different from each other. Many previous studies concerning the quantum size effects of metal films and low-dimensional materials did not consider the property of Fermi wave number or Fermi wavelength, which changes with the sizes of low-dimensional systems, so we shall indicate and emphasize that this effect of Fermi wavelength (depending on the ultra-thin metal film thickness) plays a significant role in determining the electron density of states when the film thickness is only a few times as large as the Fermi wavelength.

Now let us compare the electron densities of states in these two cases (i.e., thickness-*independent* and thickness-*dependent* Fermi wavelengths) and see at what thickness they tend to be the same. Of course, when the thickness of the metal film is very small (e.g., the metal film is composed of only a few atomic layers, e.g., the layer number $N_{\mathrm{layer}}<8$), the electron Fermi surface densities of states of these two cases are different. When the number of atomic layers is relatively large (e.g., the layer number $N_{\mathrm{layer}}\geq 8$), the electron densities of states at the Fermi surface in these two cases tend to be the same. We shall elaborate on this topic as follows.

For the first, we shall calculate the ratio of the electron Fermi surface density of states $R_{1}=\frac{\lambda_{F}^{\left(c\right)}}{2t}\left[\frac{2t}{\lambda_{F}^{\left(c\right)}}\right]=\frac{\lambda_{F}^{\left(c\right)}}{2N_{\mathrm{layer}}a}\left[\frac{2N_{\mathrm{layer}}a}{\lambda_{F}^{\left(c\right)}}\right]$ defined in Equation (20). As has been adopted in Figure 5, here we choose the (bulk) constant Fermi wavelength $\lambda_{F}^{\left(c\right)}=1.06$ nm and the single layer thickness a=0.30 nm for the ultra-thin metal film. The result for the ultra-thin metal film atomic layer numbers $N_{\mathrm{layer}}=1$∼4 is given as follows:

(23) $$\begin{array}{ccc} & & N_{\mathrm{layer}}=1,\;R_{1}=\frac{1.06\;\mathrm{nm}}{2\times 1\times 0.3\;\mathrm{nm}}\left[\frac{2\times 1\times 0.3\;\mathrm{nm}}{1.06\;\mathrm{nm}}\right]=0;\\ & & N_{\mathrm{layer}}=2,\;R_{1}=\frac{1.06\;\mathrm{nm}}{2\times 2\times 0.3\;\mathrm{nm}}\left[\frac{2\times 2\times 0.3\;\mathrm{nm}}{1.06\;\mathrm{nm}}\right]=0.883;\\ & & N_{\mathrm{layer}}=3,\;R_{1}=\frac{1.06\;\mathrm{nm}}{2\times 3\times 0.3\;\mathrm{nm}}\left[\frac{2\times 3\times 0.3\;\mathrm{nm}}{1.06\;\mathrm{nm}}\right]=0.589;\\ & & N_{\mathrm{layer}}=4,\;R_{1}=\frac{1.06\;\mathrm{nm}}{2\times 4\times 0.3\;\mathrm{nm}}\left[\frac{2\times 4\times 0.3\;\mathrm{nm}}{1.06\;\mathrm{nm}}\right]=0.883. \end{array}$$

In Appendix C, the ratios, $R_{1}$ defined in Equation (20), of the electron Fermi surface density of states for the ultra-thin metal film atomic layer numbers $N_{\mathrm{layer}}=5$∼10 are presented in Equation (A14).

We are now in a position to consider the even–odd layer oscillatory behavior of the Fermi surface electron density of states [$N^{\left(f\right)}\left(E_{F}\right)$] in the thin metal films, where the thickness-dependent Fermi wavelength $\lambda_{F}\left(N_{\mathrm{layer}}\right)$ is taken into account. We still choose the (bulk) constant Fermi wavelength $\lambda_{F}^{\left(c\right)}=1.06$ nm and the single layer thickness a=0.30 nm for the ultra-thin metal film. For the first, we consider the ratio $R_{2}=\frac{N^{\left(f\right)}\left(E_{F}\right)}{N^{\left(b\right)}\left(E_{F}\right)}$ defined in Equation (22) for the cases of film layer numbers $N_{\mathrm{layer}}=1$ and 2. From Figure 5, the layer number-dependent Fermi wavelengths $\lambda_{F}\left(N_{\mathrm{layer}}\right)$ are 0.57nm and 0.86nm, corresponding to $N_{\mathrm{layer}}=1$ and 2, respectively. The maximum number of electron half wavelength in the thickness direction (i.e., the number of Fermi half wavelengths) is $n_{\max}=\left[\frac{2N_{\mathrm{layer}}a}{\lambda_{F}\left(N_{\mathrm{layer}}\right)}\right]$ (the integer part of $\frac{2N_{\mathrm{layer}}a}{\lambda_{F}\left(N_{\mathrm{layer}}\right)}$). The result for the cases of film layer numbers $N_{\mathrm{layer}}=1$ and 2 is given by

(24) $$\begin{array}{ccc} & & N_{\mathrm{layer}}=1,\;\lambda_{F}\left(1\right)=0.57\;\mathrm{nm},\;n_{\max}=\left[\frac{2\times 1\times 0.30\;\mathrm{nm}}{0.57\;\mathrm{nm}}\right]=1,\\ & & R_{2}=\frac{1.06\;\mathrm{nm}}{2\times 1\times 0.30\;\mathrm{nm}}\times 1=1.77;\\ & & N_{\mathrm{layer}}=2,\;\lambda_{F}\left(2\right)=0.86\;\mathrm{nm},\;n_{\max}=\left[\frac{2\times 2\times 0.30\;\mathrm{nm}}{0.86\;\mathrm{nm}}\right]=1,\\ & & R_{2}=\frac{1.06\;\mathrm{nm}}{2\times 2\times 0.30\;\mathrm{nm}}\times 1=0.883. \end{array}$$

The layer number-dependent Fermi wavelength $\lambda_{F}\left(N_{\mathrm{layer}}\right)$, the number $n_{\max}$ of half Fermi wavelength and the electron density of states (expressed in terms of the ratio $R_{2}$) for the cases of film layer numbers $N_{\mathrm{layer}}=3$ and 4 are given as follows:

(25) $$\begin{array}{ccc} & & N_{\mathrm{layer}}=3,\;\lambda_{F}\left(3\right)=0.89\;\mathrm{nm},\;n_{\max}=\left[\frac{2\times 3\times 0.30\;\mathrm{nm}}{0.89\;\mathrm{nm}}\right]=2,\\ & & R_{2}=\frac{1.06\;\mathrm{nm}}{2\times 3\times 0.30\;\mathrm{nm}}\times 2=1.18;\\ & & N_{\mathrm{layer}}=4,\;\lambda_{F}\left(4\right)=0.95\;\mathrm{nm},\;n_{\max}=\left[\frac{2\times 4\times 0.30\;\mathrm{nm}}{0.95\;\mathrm{nm}}\right]=2,\\ & & R_{2}=\frac{1.06\;\mathrm{nm}}{2\times 4\times 0.30\;\mathrm{nm}}\times 2=0.883. \end{array}$$

The above characteristic quantities for the other cases corresponding to the film layer numbers $N_{\mathrm{layer}}=5$∼10 are given in Appendix C, i.e., Equations (A15)–(A17). It can be seen from the above results that with the increase in the layer number $N_{\mathrm{layer}}$ of metal films, the electron number density (expressed by the ratio $R_{2}=\frac{N^{\left(f\right)}\left(E_{F}\right)}{N^{\left(b\right)}\left(E_{F}\right)}$) exhibits an even–odd layer oscillatory effect.

**Figure 6:**
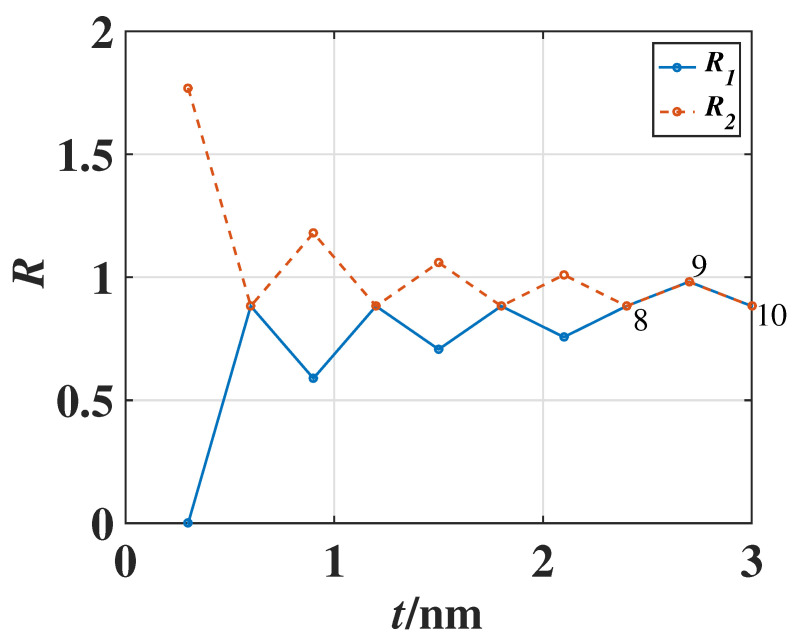
The ratios $R_{1}$ and $R_{2}$ for the Fermi surface electron densities of states in the ultra-thin metal films (with the atom layer numbers $N_{\mathrm{layer}}=1∼10$). Both of these two ratios are defined as $\frac{N^{\left(f\right)}\left(E_{F}\right)}{N^{\left(b\right)}\left(E_{F}\right)}$ with $N^{\left(f\right)}\left(E_{F}\right)$ the electron density of states in the metal *film* and $N^{\left(b\right)}\left(E_{F}\right)$ in the metal *bulk*. The effect of *dependence of the Fermi wavelength $\lambda_{F}$ on the film thickness t* is ignored for $R_{1}$, whereas such a property of *$\lambda_{F}$ depending on the film thickness t* is taken into account for $R_{2}$. When the atom layer number $N_{\mathrm{layer}}$ of the metal film is less than 8, the two parameters $R_{1}$ and $R_{2}$ exhibit different oscillatory behaviors. However, when the atom layer number $N_{\mathrm{layer}}$ of the film is more than 8, the parameters $R_{1}$ and $R_{2}$ show the same oscillatory behavior, as indicated by the points marked with the atom layer numbers “8, 9, 10" in the films. Therefore, the effect of the Fermi wavelength depending on the film thickness no longer appears when the layer number $N_{\mathrm{layer}}\geq 8$.

For convenience of comparison, the above results of the ratios $R_{1}$ and $R_{2}$ for the Fermi surface electron densities of states in the ultra-thin metal films are listed in Table 1 and the even–odd layer oscillatory behavior of $R_{1}$ and $R_{2}$ is plotted in Figure 6. Now we can draw a conclusion for this topic. Compared with the two ratios $R_{1}$ and $R_{2}$ [i.e., $\frac{N^{\left(f\right)}\left(E_{F}\right)}{N^{\left(b\right)}\left(E_{F}\right)}$ defined in Equations (20) and (22)], it can be found that when the atomic layer number $N_{\mathrm{layer}}<8$, the ratios $R_{1}$ and $R_{2}$ are different. However, when the atomic layer number $N_{\mathrm{layer}}\geq 8$, the two ratios $R_{1}$ and $R_{2}$ approach the same values, namely, only in this case of relatively large film thickness (with the layer number $N_{\mathrm{layer}}\geq 8$) can the property of the electron Fermi wavelength depending on the metal film thickness be ignored.

## 5. Discussions of Some Issues of Experimental Measurement and Theoretical Model of Quantum Size Effects

### 5.1. Quantum Size Effects in Calorimetry

Although less attention has been paid to the quantum size effect of specific heat in experiments, it would still be possible to utilize some current film growth techniques (e.g., physical deposition and epitaxial growth) for fabricating nanoscale metal films with different atomic layers, and analyze the thermodynamical characteristics by using various heat capacity measurement methods [40,41,42,43], including isothermal microcalorimetry for dynamical analysis [44]. The isothermal microcalorimetry is an efficient thermodynamical laboratory technique in real-time monitoring for studying chemical and physical processes [45,46,47].

**Figure 7:**
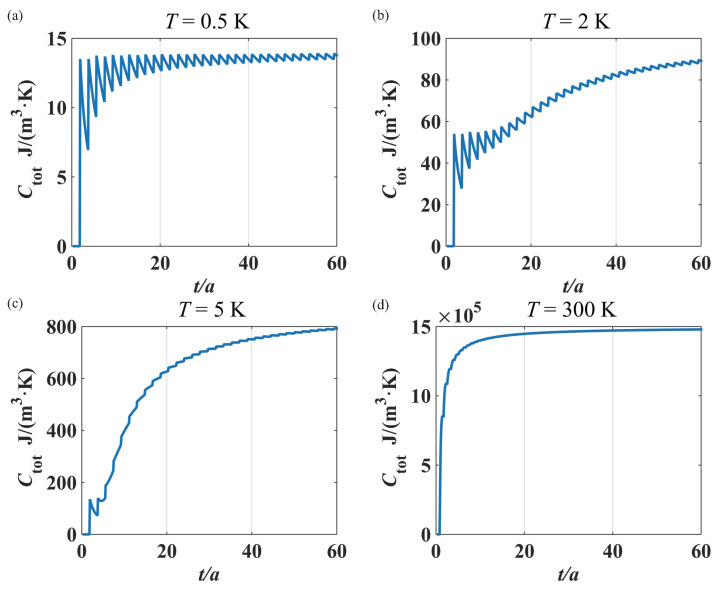
The total specific heat $C_{\mathrm{tot}}$ depending on the film thickness *t* at different temperatures *T*. (**a**) When T=0.5K, the dependance of $C_{\mathrm{tot}}$ on the atomic layer number t/a is almost the same as the electronic specific heat because the phononic specific heat decreases much more rapidly than that of electrons and can be negligibly small, and hence, one can test the quantum size effect of the electronic specific heat at this temperature. (**b**) When T=2K, the electronic and phononic specific heat would have the same order of magnitude, and both $C_{e}$ (electronic specific heat) and $C_{p}$ (phononic specific heat) could be extracted from the experimental curves $C_{\mathrm{tot}}$∼*T* at such temperatures. (**c**) When T=5K, the phononic specific heat is larger than the electronic one, i.e., the phononic specific heat can dominate in the total heat capacity at such temperatures (e.g., 5 to 100 K). (**d**) When T=300K, the electronic specific heat is negligible compared to the phononic specific heat. As the thickness of the film increases, the total specific heat of the film approaches that of the bulk material at room temperature.

Since the total heat capacity $C_{\mathrm{tot}}$ of a thin film is the sum of electronic and phononic specific heat, we have considered the even–odd layer behavior of oscillation or fluctuation for both electronic and phononic confined states in the ultra-thin film. In general, the phononic specific heat can be larger than that of electrons in a broad temperature range where the quantum statistical principles play the key roles. But at very low temperature (e.g., liquid helium temperature), they would have the same order of magnitude. When the temperature is even lower (approaching absolute zero), the phononic specific heat decreases much more rapidly than the electronic specific heat, and then the latter dominates in the total specific heat. From this fact, we can suggest that the fluctuation of phononic specific heat be tested at relatively high temperature (e.g., at 5 to 100 K), where the electronic specific heat can be negligibly small. In order for the even–odd layer fluctuation behavior of electronic specific heat to be demonstrated, we must choose extremely low temperature, such as less than 1 K, for the ultra-thin metal film. It should be pointed out that there is an alternative option for testing such a quantum size effect in the phononic and electronic specific heat. Since the dependence of electronic and phononic specific heat on temperature can be approximately characterized by $C_{e}=\gamma T$ and $C_{p}=bT^{3}$, respectively, it is possible that both $C_{e}$ and $C_{p}$ can be extracted from the experimental curves of total specific heat versus temperature ($C_{\mathrm{tot}}$∼*T*) at low temperature (e.g., lower than the Debye temperature of the metal film). Thus, the electronic and phononic contribution to the even–odd layer effects (oscillation or fluctuation as the ultra-thin film changes) can in principle be measured. Here, we present the profile of total specific heat $C_{\mathrm{total}}$ at different temperatures in Figure 7, which would provide a theoretical reference for possible experimental work. As an illustrative example, here we have chosen the Debye frequency $\omega_{D}=3.59\times 10^{13}$ $\mathrm{rad}\cdot s^{-1}$ and the average phonon velocity $\bar{c}=2750$ $m\cdot s^{-1}$. It should be noted that the phonon velocity (elastic wave speed) depends on the phonon frequency and the material temperature. As is well known, the elastic wave speed square is given by $c^{2}=\frac{\kappa }{\rho }$, where κ and ρ denote the elastic modulus and the (dynamical) mass density, respectively, of the acoustic material. Clearly, κ and ρ are the functions of the phonon frequency and the material temperature, and therefore, in the frequency integral in the specific heat of the Debye model, one needs to integrate this by piecewise approximation, where the speed of phonons takes a specific value in each frequency range. Here, for convenience, we choose a typical phonon velocity for our calculation.

Now we shall interpret in detail the two characteristics in the specific heat curves in Figure 7, where the jagged ripples become weaker as the film thickness *t* and the temperature *T* increase.

For the first, we elucidate the thickness-related behavior in the specific heat. In the lattice specific heat (11) of the ultra-thin metal film, there is the factor $\frac{1}{t}\left[\frac{\omega t}{\pi \bar{c}}\right]$ related to the film thickness *t*. In this factor, if $n<\frac{\omega t}{\pi \bar{c}}<n+1$, i.e., $n\frac{\pi \bar{c}}{\omega }<t<\left(n+1\right)\frac{\pi \bar{c}}{\omega }$, one can have the integer $\left[\frac{\omega t}{\pi \bar{c}}\right]=n$. Thus, we can obtain the following relation: $\frac{n}{n+1}\cdot \frac{\omega }{\pi \bar{c}}<\frac{n}{t}<\frac{\omega }{\pi \bar{c}}$. Therefore, the thickness-related factor $\frac{1}{t}\left[\frac{\omega t}{\pi \bar{c}}\right]$ is in the range

(26) $$\begin{array}{c} \frac{n}{n+1}\cdot \frac{\omega }{\pi \bar{c}}<\frac{1}{t}\left[\frac{\omega t}{\pi \bar{c}}\right]<\frac{\omega }{\pi \bar{c}}. \end{array}$$

Since $n<\frac{\omega t}{\pi \bar{c}}<n+1$, the integer $n=\left[\frac{\omega t}{\pi \bar{c}}\right]$ grows as the film thickness *t* increases. When the film thickness *t* is adequately large, both $\left[\frac{\omega t}{\pi \bar{c}}\right]$ and $\frac{n}{n+1}\cdot \frac{\omega }{\pi \bar{c}}$ increase with *t*, and finally, in the 3-D bulk limit, $\frac{1}{t}\left[\frac{\omega t}{\pi \bar{c}}\right]$ approaches $\frac{\omega }{\pi \bar{c}}$. As a result, the jagged ripple amplitude in its curve of $\frac{1}{t}\left[\frac{\omega t}{\pi \bar{c}}\right]$ can be characterized by

(27) $$\begin{array}{c} \Delta \left(\frac{1}{t}\left[\frac{\omega t}{\pi \bar{c}}\right]\right)=\left(1-\frac{n}{n+1}\right)\frac{\omega }{\pi \bar{c}}=\frac{1}{n+1}\cdot \frac{\omega }{\pi \bar{c}}. \end{array}$$

This, therefore, means that the larger the thickness *t* is, the smaller the jagged ripple amplitude (27) is. As a result, in a 3-D bulk metal, such a quantum size effect (jagged ripples) in the specific heat disappears. The above property can also be found in Figure 3, Figure 4 and Figure 7, where the jagged ripple amplitudes become weaker when the thickness *t* increases.

We shall now elaborate on the second property in the thin film specific heat curves in Figure 7, where the jagged ripples become weaker as the temperature *T* increases. In the Debye model for a bulk material, the lattice specific heat $C_{p}^{\left(b\right)}$ given in Equation (12) can be rewritten as

(28) $$\begin{array}{ccc} C_{p}^{\left(b\right)} & = & \frac{3k_{B}}{2\pi^{2}{\bar{c}}^{3}}\int_{0}^{\omega_{D}}\frac{{\left(\frac{\hbar \omega }{k_{B}T}\right)}^{2}\exp\left(\frac{\hbar \omega }{k_{B}T}\right)}{{\left[\exp\left(\frac{\hbar \omega }{k_{B}T}\right)-1\right]}^{2}}\omega^{2}d\omega \\ & = & \frac{3k_{B}}{2\pi^{2}{\bar{c}}^{3}}{\left(\frac{k_{B}T}{\hbar }\right)}^{3}\int_{0}^{x_{D}}\frac{x^{4}e^{x}}{{\left(e^{x}-1\right)}^{2}}dx, \end{array}$$

where $x=\frac{\hbar \omega }{k_{B}T}$ and $x_{D}=\frac{\hbar \omega_{D}}{k_{B}T}$. We shall analyze the maximum of the integrand $F\left(x\right)=\frac{x^{4}e^{x}}{{\left(e^{x}-1\right)}^{2}}$ and find the phonon frequency of the modes that play the significant role in the contribution to the lattice specific heat. The derivative of F(x) with respect to *x* is given by

(29) $$\begin{array}{c} \frac{dF(x)}{dx}=\frac{x^{3}e^{x}\left(4e^{x}-4-xe^{x}-x\right)}{{\left(e^{x}-1\right)}^{3}}. \end{array}$$

Let $\frac{dF(x)}{dx}=0$, and we have $e^{x}=\frac{4+x}{4-x}$. This equation can be solved by using the intersection of two curves $y_{1}=e^{x}$ and $y_{2}=\frac{4+x}{4-x}$. The solution is x=A with 3.5<A<4 and the numerical factor F in the phononic specific heat takes the maximum when $\omega =A\frac{k_{B}T}{\hbar }$ at the temperature *T*. Then, it can be said that the phonon modes around the frequency $\omega =A\frac{k_{B}T}{\hbar }$ make the significant contribution to the phononic specific heat in a bulk material.

As far as the specific heat of a thin film is concerned, the thickness-related integer $\left[\frac{\omega t}{\pi \bar{c}}\right]=\left[A\frac{k_{B}T}{\hbar }\frac{t}{\pi \bar{c}}\right]$ corresponding to the phonon mode of maximum contribution to the film specific heat will increase from a first integer (say, *m*) to a second one (say, m+1) as the temperature *T* rises. Thus, the relative amplitude of jagged ripples is $\frac{1}{m+1}$, which becomes smaller or approaches almost zero at higher temperature, because the integer *m* is large at higher temperature *T*. This characteristic behavior can be found in Figure 7, where the ripple amplitudes decrease as the temperature *T* increases.

Based on the above result, we shall then include two tables for comparing the specific heat values across different temperatures and provide a detailed discussion in order to analyze the temperature-dependent behavior of the specific heat of thin films.

The numerical values of the temperature-dependent total specific heat $C_{\mathrm{tot}}$ of thin metal films with different thicknesses *t* (t/a is the atom layer number of the thin film) are presented in Table 2 at the absolute temperature T=0.5∼4.5 K and Table 3 at the temperature T=5.0∼10.0 K. In Table 2, the data show that, when the temperature increases, the total specific heat also increases significantly for all film thicknesses (e.g., the layer number t/a=10,20, and 30). It can be found that the thicker films (e.g., t/a=30) exhibit a slightly higher specific heat compared with the thinner films (e.g., t/a=10) at the same temperature. This indicates that both the temperature and the film thickness influence the specific heat and that the thicker films exhibit a more pronounced increase in specific heat (with a rise in temperature) than the thinner ones. In Table 3, the data show that the total specific heat rises sharply for all film thicknesses. Notably, the thicker films (e.g., t/a=30) consistently have a higher specific heat compared with the thinner ones (e.g., t/a=10) at the same temperature. The rate of increase in the specific heat with temperature is more obvious in the thicker films, i.e., the thicker films exhibit more significant sensitivity to the temperature change than the thinner films.

### 5.2. Fermi Energy Correction in Quantum Size Effects at Finite Temperature

In the literature [48], Balcerzak has studied the thickness-dependent Fermi wave number and Fermi surface state density oscillation at the absolute temperature T=0 in thin films, where his idea and model were very clear and the problem was analyzed and calculated in great detail [48]. In what follows, by modifying the result (17), which is applicable only at T=0, we shall give a procedure for calculating the electron Fermi energy in a metal film at finite temperature. For the first, within the framework of quantum statistics, the total number of free electrons in a thin metal film takes the form

(30) $$\begin{array}{ccc} N_{\mathrm{tot}} & = & g_{s}\sum_{k}\frac{1}{\exp(\frac{\varepsilon_{k}-\mu }{k_{B}T})+1}\\ & = & g_{s}\sum_{k_{z}}\frac{1}{{\left(2\pi \right)}^{2}}\int dxdy\int_{k_{⊥}}\frac{dk_{x}dk_{y}}{\exp\left(\frac{\varepsilon_{k}-\mu }{k_{B}T}\right)+1}\\ & = & g_{s}\sum_{k_{z}}\frac{S}{{\left(2\pi \right)}^{2}}\int_{k_{⊥}}\frac{\pi dk_{⊥}^{2}}{\exp\left(\frac{\varepsilon_{k}-\mu }{k_{B}T}\right)+1}, \end{array}$$

where the electron kinetic energy is $\varepsilon_{k}=\frac{\hbar^{2}k^{2}}{2m^{*}}$ with $k^{2}=k_{z}^{2}+k_{⊥}^{2}={\left(\frac{n\pi }{t}\right)}^{2}+\left(k_{x}^{2}+k_{y}^{2}\right)$ and the volume element $dk_{x}dk_{y}$ in the wave number space is $dk_{x}dk_{y}=\pi dk_{⊥}^{2}=\pi d\left[k^{2}-{\left(\frac{n\pi }{t}\right)}^{2}\right]$. The free electron number $N_{\mathrm{tot}}$ can be rearranged as

(31) $$\begin{array}{ccc} N_{\mathrm{tot}} & = & g_{s}\sum_{n=1}^{\infty }\frac{S}{{\left(2\pi \right)}^{2}}\pi \int_{k\geq \frac{n\pi }{t}}^{\infty }\frac{d[k^{2}-{\left(\frac{n\pi }{t}\right)}^{2}]}{\exp\left(\frac{\varepsilon_{k}-\mu }{k_{B}T}\right)+1}\\ & = & g_{s}\sum_{n=1}^{\infty }\frac{S}{{\left(2\pi \right)}^{2}}\pi [\int_{k=0}^{\infty }\frac{dk^{2}}{\exp(\frac{\frac{\hbar^{2}k^{2}}{2m^{*}}-\mu }{k_{B}T})+1}\\ & & -\int_{k=0}^{\frac{n\pi }{t}}\frac{dk^{2}}{\exp(\frac{\frac{\hbar^{2}k^{2}}{2m^{*}}-\mu }{k_{B}T})+1}], \end{array}$$

which can be rewritten as

(32) $$\begin{array}{ccc} N_{\mathrm{tot}} & = & \frac{g_{s}\pi S}{{\left(2\pi \right)}^{2}}\sum_{n=1}^{\infty }\left[G\left(k^{2},\mu ,k_{B}T\right){|}_{k=0}^{\infty }-G\left(k^{2},\mu ,k_{B}T\right){|}_{k=0}^{\frac{n\pi }{t}}\right], \end{array}$$

where $G(k^{2},\mu ,k_{B}T)$ is the integral result for $\int \frac{dk^{2}}{\exp\left(\frac{\frac{\hbar^{2}k^{2}}{2m^{*}}-\mu }{k_{B}T}\right)+1}$. We define the free electron number density (per unit volume) $n_{e}=\frac{N_{\mathrm{tot}}}{St}$ and, from Equation (32), we can obtain

(33) $$\begin{array}{c} n_{e}=\frac{g_{s}\pi }{{\left(2\pi \right)}^{2}t}\sum_{n=1}^{\infty }\left[G\left(k^{2},\mu ,k_{B}T\right){|}_{k=0}^{\infty }-G\left(k^{2},\mu ,k_{B}T\right){|}_{k=0}^{\frac{n\pi }{t}}\right]. \end{array}$$

For a given thin film, the free electron number density $n_{e}$ can be identified as a known physical quantity, and then the chemical potential (Fermi energy) μ is a function of the film thickness *t* and the absolute temperature *T*, i.e., μ=μ(t,T). It can be found that the chemical potential μ(t,T) can in principle be solved based on the above Equation (33), and as a result, based on Equation (33), the Fermi wave number $k_{F}$ and Fermi wavelength $\lambda_{F}$ that depend on the thin film thickness *t* or on the atom layer number $N_{\mathrm{layer}}$ at the nonzero absolute temperature can be derived through the relations $\mu =\frac{\hbar^{2}k_{F}^{2}}{2m^{*}}$ and $\lambda_{F}=\frac{2\pi }{k_{F}}$.

### 5.3. Various Quantum Size Effects in Solid State Physics and Quantum Gas Statistics

The size-dependent behavior of electronic and lattice specific heat of an ultra-thin metal film has been considered, and the effect of “*even–odd layer resonant oscillation*” depending on the film thickness has been studied in this paper. In an ultra-thin metal film or nanofilm, which is only several atomic layers thick, the electrons confined in the thickness direction are quantized into discrete energy levels, forming the quantum well states [12]. On account of quantum confinement in the vertical dimension of the ultra-thin metal film, the quantum size effect plays an important role in determining the electronic and magnetic properties, thermodynamics, and electron kinetics in thin metal films. An oscillatory curve with a damped amplitude in these physical quantities appears in such ultra-thin films. In solid state physics, there are a number of properties that depend on the electron density of states $N(E_{F})$, which can exhibit the even–odd layer oscillatory effect. For example, Pauli spin paramagnetic susceptibility in a metal is $\chi =\mu_{B}^{2}N\left(E_{F}\right)$ (based on a local electron exchange model) or $\chi =\mu_{B}^{2}N(E_{F})/\left[1-\frac{UN(E_{F})}{2}\right]$ (based on energy band theory) with $\mu_{B}$ the Bohr magneton and *U* the interaction potential energy between spin-up and spin-down electrons [49]. The Coulomb shielding potential of electron gas in a metal is given by $\varphi \left(r\right)=-\frac{e}{4\pi \varepsilon_{0}r}\exp\left[-e\sqrt{\frac{N(E_{F})}{\varepsilon_{0}}}r\right]$; The metal direct current conductivity is $\sigma =\frac{2\pi q^{2}\hbar^{3}}{m^{2}}{\left|D\right|}_{\mathrm{av}}^{2}{\left(N(E_{F})\right)}^{2}$, with ${\left|D\right|}_{\mathrm{av}}^{2}$ being the electron average transition matrix elements in the photon absorption process [38,49]. All these effects are related intimately to the density of electron states $N(E_{F})$ near the Fermi level. Therefore, it is readily predicted that these quantum size effects would emerge in solid state physics. Clearly, the paramagnetic susceptibility, Coulomb shielding potential, and direct current conductivity in ultra-thin metal films will also have oscillatory dependence on the thickness of nanoscale metal films.

Quantum gases in a thin cavity with a thickness with order of magnitude of gaseous particle wavelength can also exhibit the quantum size effect. As an illustrative example, we shall calculate the energy density of a thermal radiation gas (thermal photon state) in a 2-D high-quality thin cavity, where the thickness is in the *z*-direction and the cavity sides in the *x*-*y* plane are large enough (identified as infinitely long dimensions). Similar to the formula of mode number given in Equation (17), the photon mode number is $N_{\mathrm{mode}}\left(\omega \right)=g_{s}\sum_{n=1}^{n_{\max}}\frac{S}{{\left(2\pi \right)}^{2}}\pi \left[{\left(\frac{\omega }{c}\right)}^{2}-{\left(\frac{n\pi }{t}\right)}^{2}\right]$ with $g_{s}=2$ (the photon spin or polarization degree of freedom) and $n_{\max}=\left[\frac{\omega t}{\pi c}\right]$ (the maximum number of half wavelength). Then, the photon density of states in the present thin cavity can be determined by $g_{\mathrm{photon}}\left(\omega \right)=\frac{1}{St}\frac{\partial N_{\mathrm{mode}}\left(\omega \right)}{\partial \omega }=\sum_{n=1}^{n_{\max}}\frac{\omega }{\pi c^{2}}\frac{1}{t}=\frac{\omega }{\pi c^{2}}\frac{1}{t}n_{\max}$, i.e., the result is given by

(34) $$\begin{array}{c} g_{\mathrm{photon}}\left(\omega \right)=\frac{\omega }{\pi c^{2}}\frac{1}{t}\left[\frac{\omega t}{\pi c}\right]. \end{array}$$

When the thickness t→+∞, the density of states of thermal photons in the thin cavity can be rewritten as $g_{\mathrm{photon}}\left(\omega \right)\to \frac{\omega }{\pi c^{2}}\frac{1}{t}\cdot \frac{\omega t}{\pi c}=\frac{\omega^{2}}{\pi^{2}c^{3}}$, which is the well-known photon density of states in the 3-D space. Now the energy density of the thermal radiation gas in the thin cavity is $\rho_{\varepsilon }=\int_{0}^{\infty }\frac{\hbar \omega }{\exp(\frac{\hbar \omega }{k_{B}T})-1}g_{\mathrm{photon}}\left(\omega \right)d\omega$. It can be expected that such an energy density will show the jagged ripples in its curve as the thickness *t* increases and finally approach the photon gas energy density in the 3-D space.

In addition to the photon gas, the quantum size effect can also be exhibited by a Boson atomic gas in a thin cavity. In the mode number, $N_{\mathrm{mode}}=\sum_{n=1}^{n_{\max}}\frac{S}{{\left(2\pi \right)}^{2}}\pi \left[k^{2}-{\left(\frac{n\pi }{t}\right)}^{2}\right]$, of de Broglie atomic matter waves, it can be written as $N_{\mathrm{mode}}\left(\varepsilon \right)=\sum_{n=1}^{n_{\max}}\frac{S}{{\left(2\pi \right)}^{2}}\pi \left[\frac{2m\varepsilon }{\hbar^{2}}-{\left(\frac{n\pi }{t}\right)}^{2}\right]$ with $n_{\max}=\left[\frac{kt}{\pi }\right]$, where $\hbar^{2}k^{2}=2m\varepsilon$. Then, the Bosonic gas density of states is given by $g_{\mathrm{Boson}}\left(\varepsilon \right)=\frac{1}{St}\frac{\partial N_{\mathrm{mode}}\left(\varepsilon \right)}{\partial \varepsilon }=\sum_{n=1}^{n_{\max}}\frac{2\pi m}{{\left(2\pi \hbar \right)}^{2}}\frac{1}{t}=\frac{2\pi m}{{\left(2\pi \hbar \right)}^{2}}\frac{1}{t}\left[\frac{kt}{\pi }\right]$, i.e., it can be rewritten as

(35) $$\begin{array}{c} g_{\mathrm{Boson}}\left(\varepsilon \right)=\frac{2\pi m}{{\left(2\pi \hbar \right)}^{2}}\frac{1}{t}\left[\frac{{\left(2m\varepsilon \right)}^{\frac{1}{2}}t}{\pi \hbar }\right]. \end{array}$$

When the thin cavity thickness *t* increases, in the limit of t→∞, the Bosonic atomic gas density of states is $g_{\mathrm{Boson}}\left(\varepsilon \right)\to \frac{2\pi m}{{\left(2\pi \hbar \right)}^{2}}\frac{1}{t}\cdot \frac{{\left(2m\varepsilon \right)}^{\frac{1}{2}}t}{\pi \hbar }=\frac{2\pi }{{\left(2\pi \hbar \right)}^{3}}{\left(2m\right)}^{\frac{3}{2}}\varepsilon^{\frac{1}{2}}$, which is the well-known density of states of non-relativistic Bosons in the 3-D space. In the 2-D thin cavity, the Bosonic particle number density is given by $n_{\mathrm{Boson}}=\int_{\mu }^{\infty }\frac{1}{\exp(\frac{\varepsilon -\mu }{k_{B}T})-1}g_{\mathrm{Boson}}\left(\varepsilon \right)d\varepsilon$, where μ denotes the chemical potential, and the Bose–Einstein condensation phase transition (critical) temperature $T_{c}$, where the chemical potential tends to μ=0, is determined through the relation $n_{\mathrm{Boson}}=\int_{0}^{\infty }\frac{1}{\exp(\frac{\varepsilon }{k_{B}T_{c}})-1}g_{\mathrm{Boson}}\left(\varepsilon \right)d\varepsilon$ (for the non-relativistic atomic gas). In a fashion similar to the above quantum size effects, the jagged ripples would also be exhibited in the curve of the Bose–Einstein condensation critical temperature $T_{c}$ as the thin cavity thickness *t* increases.

In the present paper, we have used the method relevant to the so-called *hard-wall boundary condition*, which is a rather simple model, because the films with only very few atomic layers cannot be identified as infinitely deep potential wells and the confined electronic states will unavoidably spread out in the thickness direction. Since the electrons can move towards the solid surface and then be reflected into the medium bulk, the extended electron’s quantum mechanical wave functions in the film materials are the states whose probability densities exponentially decay into vacuum [50]. Though, as is known, such evanescent components in the wave functions of electronic states are small, it may still affect the numerical results of the electronic specific heat of the nanometer-sized metal films in the present paper. The electronic states in the thin films used in the paper have been simplified, but it can still exhibit the fundamental physical principle of quantum size effects; specifically, for those films with more than three or four atomic layers, the wave function extension effect (on the hard-wall boundary) would diminish as the film thickness increases, and in this case, such an undesirable effect (wave function extension) could be ignored in the even–odd layer oscillatory behavior of electronic specific heat. If, however, we need to achieve more accurate results of quantum size effects, in particular when the ultra-thin film has very few atomic layers, we must resort to a theoretical formulation of electronic states of finite-size crystals. As is known, the solid surface breaks the crystal 3-D periodicity, and the electronic states in the neighborhood of surface differ much from of the Bloch waves in an infinite-size crystal [50]. There is such an analytical method for treating the effects of quantum confinement of electronic Bloch waves in crystals of finite size and lower dimensions [51]. In this reference [51], in order to show how the electronic states extend in the finite-size crystals, where the periodic boundary conditions and spatial translation invariance no longer hold true, the author made an effort to establish a theoretical framework for the confined electronic states of finite-size crystals. In order to theoretically demonstrate the present quantum size effect and to compare it with the practical measurements of specific heat, this analytical method for crystals of finite size [51] deserves consideration for this subject. Other methods, which have been employed in surface physics, e.g., jellium model, tight-binding approximation, muffin-tin approximation, as well as pseudo-potential method [50], could also be applicable to the topic in the present paper.

## 6. Physical Novelty and Potential Applications of Quantum Size Effects of Nanoscale Structures in Calorimetry

In this paper, regarding the quantum size correction to the specific heat of nanoscale films, we choose metal materials. The choice of metal for this study, as opposed to insulators such as ceramic materials, is primarily due to the unique electronic and phononic properties of metals that are significantly influenced by quantum size effects, particularly in ultra-thin films. Here are some reasons for why metals are chosen in this work:

(i) *Electron confinement effect and standing wave density of states*: Metals have a high density of free electrons, which makes them highly sensitive to quantum confinement effects. When the thickness of a metal film is reduced to the nanoscale, the electronic states become quantized, leading to oscillatory behaviors in properties like specific heat. The density of states at the Fermi level, which is crucial in determining the specific heat and other electronic properties, changes significantly with the film thickness in metals. This quantum size effect is less pronounced or behaves differently in other solids such as ceramic materials, which typically have a different electronic structure with fewer free electrons.
(ii) *Metallic bonding and conductivity*: The metallic bonding in metals allows for a free electron gas model, where electrons can move relatively freely throughout the material. This property makes metals more suitable for studying how the electronic specific heat and other thermodynamic properties are affected by film thickness at the quantum scale. In contrast, ceramics generally have ionic or covalent bonding, leading to a different set of thermal and electronic properties that do not exhibit the same degree of quantum size effects.
(iii) *Phonon contributions*: In metals, the contribution of phonons (lattice vibrations) to specific heat also exhibits quantum size effects due to the confinement of these vibrations in the thin film. While in ceramics, phonons do make a contribution to their thermal properties, the overall behavior of this contribution differs from metals because ceramics often have more complex crystal structures and bonding types, leading to different phonon dispersion relations.
(iv) *Technological applications*: Metals are commonly used in electronic and photonic devices, where ultra-thin films are often employed. Understanding the quantum size effects in metal films is crucial for developing new technologies in these fields. The study findings have direct implications for the design of nanoscale devices that rely on the unique electronic properties of metals.

In summary, the choice of metals over ceramic materials is due to the significant quantum size effects on the electronic and phononic properties of metals, which are more pronounced and critical in understanding the thermodynamics of ultra-thin films. These effects are less relevant or manifest differently in ceramics, making metals the more appropriate material for this study.

We shall also discuss some physical significance and application-oriented aspects of quantum size effects in calorimetry for this study.

(i) *Quantum size correction to specific heat*: The present study specifically focuses on the quantum size effect on both electronic and phononic specific heat in ultra-thin metal films. Though the concept of quantum size effects is well known in low-dimensional materials, the detailed investigation into the oscillatory behavior of specific heat as a function of the film size would add insights that are critical for understanding and designing nanoscale devices.
(ii) *Size-dependent electronic Fermi wavelength*: A significant novelty in this study lies in the explicit modeling of the size-dependent variation in the electron Fermi wavelength in ultra-thin films. This aspect has often been overlooked in previous studies, where the Fermi wavelength was typically treated as a constant. We provide an analytic formulation that demonstrates how the Fermi wavelength changes with the film thickness, leading to resonant oscillations in specific heat, which is crucial for accurate predictions in calorimetry at the nanoscale.
(iii) *Theoretical framework for experimental applications*: The present paper presents a theoretical framework that may be applied directly to the design and optimization of nanoscale thermodynamical devices. By establishing the relationship between thermodynamical properties and film size, we offer a practical guide for experimentalists aiming to exploit quantum size effects in real-world applications such as superconducting films, thermoelectric materials, and quantum well structures.
(iv) *Comprehensive analysis of both electronic and phononic contributions*: Unlike some studies that focus solely on either electronic or phononic effects, our research provides a comprehensive analysis of both contributions to specific heat in ultra-thin films. The dual consideration of these effects and their interplay would represent an approach to understanding thermal properties at the nanoscale.
(v) *Potential for new experimental techniques*: We also discuss the potential applications for new experimental techniques based on the theoretical findings, such as the use of specific heat measurements to detect quantum size effects in ultra-thin films. This application-oriented perspective is novel and may suggest new directions for experimental research that has not been fully explored.

The modeling of quantum size effects in ultra-thin metal films, as presented in this study, would open up several promising perspectives for applications across various advanced technologies. Here are some key areas where the modeling for quantum size effects could be particularly impactful:

(i) *Nanoscale electronic devices such as quantum well structures*: The understanding of quantum confinement in ultra-thin metal films could be directly applied to the design of quantum well structures in nanoscale electronic devices. This would be particularly relevant for transistors, sensors, and other components where precise control over electronic properties is essential. This can be used in thermal management; specifically, the oscillatory behavior of specific heat due to quantum size effects could be leveraged to develop materials with tailored thermal properties. This is critical in microelectronics, where managing heat at the nanoscale is a major challenge.
(ii) *Superconducting nanodevices*: The insights gained from this modeling can be applied to the design of superconducting films and nanowires. When physicists understand how specific heat and other thermodynamic properties oscillate with film thickness, it can be expected that this mechanism would offer potential help in optimizing or controlling superconducting transition temperatures and enhancing certain performance of superconducting circuits used in sensitive magnetic sensors and possible quantum computing devices.
(iii) *Energy storage and conversion*: The principles from this modeling could assist the design of thin film batteries, particularly where the electrode materials are metallic. By manipulating the thickness and layering of these films, it may be possible to enhance energy storage capacities and charge/discharge rates.
(iv) *Thermoelectric materials*: The ability to control and predict thermal properties at the nanoscale could contribute to the development of advanced thermoelectric materials. These materials may convert heat into electricity (or vice versa) more efficiently if the specific heat and thermal conductivity are optimized through the understanding of quantum size effects.
(v) *Metamaterials and plasmonics*: The modeling can aid in the design of plasmonic and optoelectronic devices, where controlling the behavior of electrons and phonons at the nanoscale is crucial for manipulating light at sub-wavelength scales. This would have applications in sensors, imaging, and communication technologies. It can be used in Surface Plasmon Resonance, namely, ultra-thin metal films are often used in plasmonic applications, where surface plasmon resonance is a key feature. The ability to model and predict changes in electron density of states can help in fine-tuning these resonances for improved sensitivity in biosensors and other analytical tools.
(vi) *Fundamental physics and material science*: The modeling in the present work would provide a framework for exploring fundamental quantum phenomena and physical principles in lower dimensions. It could guide some experimental calorimetry studies aiming to observe and harness quantum size effects in lower-dimensional materials and structures, potentially leading to the discovery of novel physical properties and phenomena as well as possible states of matter. We expect that the insights from this modeling could be used to design new materials and structures with customized electronic and thermal properties, which may be critical in various high-tech applications, e.g., the nanoscale sensors that are sensitive to geometric shape and size.

In conclusion, we expect that the application of this modeling of quantum size effects in ultra-thin metal films may extend across a broad range of fields, from electronics and energy to possible areas of fundamental science and material engineering.

## 7. Concluding Remarks

We shall make a few remarks concerning the ultra-thin film thickness dependence of physical properties of metals. We interpret why the specific heat resulting from state density distribution of both electrons and phonons in a metallic nanofilm depends (in an oscillatory fashion) on its thickness: When the thickness of the ultra-thin metal film is exactly an integer multiple of half wavelength of the standing wave of electrons in the thickness direction, the corresponding density of states would become maximized, and the electronic specific heat takes its maximum. If, however, the thickness of the metal film is not the integer times of the half wavelength of electrons in the thickness direction, the specific heat decreases, falling to a minimum. and when the film thickness increases, the electronic specific heat will immediately jump to its new maximum, because the increasing thickness becomes again a new integer multiple of the half wavelength of electron standing wave mode, and this leads to a sudden change in the electron density of states, as is shown in Figure 2. Therefore, the electron density of states exhibits a characteristic of *stepwise decrease and sudden jump* when the film thickness increases, and leads to the oscillatory behavior of the relevant measurable physical parameters. As the film thickness increases, the oscillation will become weaker, and the influence of quantum size effect on the measurable physical quantities is suppressed. If the thin film thickness is sufficiently large, the size-dependent oscillation almost disappears and the electronic and lattice specific heat of ultra-thin metal films will approach that of bulk metals.

It should be pointed out that in almost all the work of quantum size effects of various physical properties in thin films, including superconducting critical temperature [12], film electric conductivity [13,14], metallic work function [15], magnetoresistance [13], film Hall effect [13,16], and low-energy electron absorption in metal layers [17], the film Fermi wavelength of electrons was identified as constant numbers, i.e., the size or shape dependence of Fermi wavelength has never been taken into account. This result is valid for a thin film with sufficiently large thickness. For the ultra-thin film, however, this might no longer hold true. We have shown in this paper that the film Fermi wavelength in a thin metal film with thickness less than ten atomic monolayers can vary drastically with the film thickness. Such a dramatic change can also occur for lattices or photons, e.g., the Debye frequency of film lattices also depends on the ultra-thin film thickness. The behavior of layer number-dependent electron Fermi wavelength in the thin metal film at the absolute temperature T=0 has been treated. We also generalized this effect to the case T≠0 and gave a formal theoretical model for treating the electron Fermi wavelength that depends on the film thickness or atom layer number of thin film.

## Figures and Tables

**Table 1:** The ratios $R_{1}$ and $R_{2}$ for the electron densities of states.

$N_{\mathrm{layer}}$	1	2	3	4	5	6	7	8	9	10
** $R_{1}$ **	0	0.883	0.589	0.883	0.707	0.883	0.757	0.883	0.981	0.883
** $R_{2}$ **	1.77	0.883	1.18	0.883	1.06	0.883	1.01	0.883	0.981	0.883

**Table 2:** The temperature-dependent total specific heat $C_{\mathrm{tot}}$ of the films with layer numbers t/a=10,20,30 at T=0.5∼4.5 K. The unit of the specific heat $C_{\mathrm{tot}}$ in this table is $J/\left(m^{3}\cdot K\right)$.

	0.5 K	1.0 K	1.5 K	2.5 K	3.5 K	4.5 K
$C_{\mathrm{tot}}\left(t/a=10\right)$	12.8	25.7	38.5	67.7	130.7	278.5
$C_{\mathrm{tot}}\left(t/a=20\right)$	12.8	25.7	40.4	97.8	226.4	460.0
$C_{\mathrm{tot}}\left(t/a=30\right)$	13.7	27.9	47.1	121.0	270.7	531.2

**Table 3:** The temperature-dependent total specific heat $C_{\mathrm{tot}}$ of the films with layer numbers t/a=10,20,30 at T=5.0∼10.0 K. The unit of the specific heat $C_{\mathrm{tot}}$ in this table is $J/\left(m^{3}\cdot K\right)$.

	5.0 K	6.0 K	7.0 K	8.0 K	9.0 K	10.0 K
$C_{\mathrm{tot}}\left(t/a=10\right)$	397.4	746.7	1272.0	2007.2	2986.8	4245.8
$C_{\mathrm{tot}}\left(t/a=20\right)$	627.0	1083.4	1732.4	2609.0	3748.7	5186.5
$C_{\mathrm{tot}}\left(t/a=30\right)$	714.0	1206.6	1898.0	2823.4	4018.0	5517.1

## Data Availability

Our manuscript is a purely theoretical work. Throughout the course of this research, we did not collect or process any experimental data. As such, no new data were created or analyzed in this study.

## Appendix A. Electronic Specific Heat of a Metal

For readers’ convenience, we shall review the theoretical formalism for the electronic specific heat of a metal [39,49,52]. The total internal energy *U* of electrons in this metal is given by $U=\int_{0}^{+\infty }Ef(E)N(E)dE$, where $f\left(E\right)=\frac{1}{e^{\left(E-E_{F}\right)/\left(k_{B}T\right)}+1}$ is the Fermi–Dirac distribution and N(E) is the electron density of states. One can define a function $R\left(E\right)=\int_{0}^{E}EN(E)dE$, which stands for the total internal energy when the quantum states below the energy eigenvalue *E* is filled by electrons. Integration by parts of the internal energy function *U* yields

(A1) $$U={\left.f(E)R(E)\right|}_{0}^{+\infty }-\int_{0}^{+\infty }R\left(E\right)\frac{\partial f}{\partial E}dE.$$

Obviously, the first term on the right-handed side of Equation (A1) vanishes because when E=+∞, the Fermi–Dirac distribution f(E) is zero, and when E=0, the function R(E) vanishes. Note that the function $-\frac{\partial f}{\partial E}$ is very steep with its maximum at $E=E_{F}$. Thus, $-\frac{\partial f}{\partial E}$ differs significantly from zero near the Fermi level (in the energy range of the order of $k_{B}T$ around the Fermi energy level $E_{F}$). Then, one can extend the lower limit of integral to a negative infinity, i.e., $U\to \int_{-\infty }^{+\infty }R\left(E\right)\left(-\frac{\partial f}{\partial E}\right)dE$. Expanding R(E) as the Taylor series around $E_{F}$, we can obtain

(A2) $$R\left(E\right)=R\left(E_{F}\right)+R^{\prime }\left(E_{F}\right)\left(E-E_{F}\right)+\frac{1}{2}R^{\prime \prime }\left(E_{F}\right){\left(E-E_{F}\right)}^{2}+\cdot \cdot \cdot$$

By using the idea and method of statistical thermodynamics in solid state physics [39,49,52], substitution of Equation (A2) into the internal energy *U* yields $U=R\left(E_{F}\right)+\frac{\pi^{2}}{6}R^{\prime \prime }\left(E_{F}\right){\left(k_{B}T\right)}^{2}$. Expanding the first term of this equation around the Fermi energy $E_{F}^{0}$ at absolute zero temperature, one can obtain [39,49,52]

(A3) $$U=R\left(E_{F}^{0}\right)+R^{\prime }\left(E_{F}^{0}\right)\left(E_{F}-E_{F}^{0}\right)+\frac{\pi^{2}}{6}R^{\prime \prime }\left(E_{F}^{0}\right){\left(k_{B}T\right)}^{2}.$$

Here, the Fermi energy depending on the temperature can take the form [49]:

(A4) $$E_{F}=E_{F}^{0}\left\{1-\frac{\pi^{2}}{6E_{F}^{0}}{\left[\frac{d}{dE}\ln N\left(E\right)\right]}_{E_{F}^{0}}{\left(k_{B}T\right)}^{2}\right\}.$$

Then, by substituting Equation (A4) into Equation (A3), the total internal energy *U* appears to be of the form

(A5) $$U=R\left(E_{F}^{0}\right)+\frac{\pi^{2}}{6}R^{\prime }\left(E_{F}^{0}\right){\left(k_{B}T\right)}^{2}\left\{-{\left[\frac{d}{dE}\ln N\left(E\right)\right]}_{E_{F}^{0}}+{\left[\frac{d}{dE}\ln R^{\prime }\left(E\right)\right]}_{E_{F}^{0}}\right\}.$$

From the definition of R(E), one can obtain $R^{\prime }\left(E\right)=EN\left(E\right)$, and then we have

(A6) $$\begin{array}{c} -\frac{d\ln N(E)}{dE}+\frac{d\ln R^{\prime }\left(E\right)}{dE}=\frac{d\ln E}{dE}=\frac{1}{E}. \end{array}$$

Now Equation (A5) can be rewritten as

(A7) $$U=R\left(E_{F}^{0}\right)+\frac{\pi^{2}}{6}N\left(E_{F}^{0}\right){\left(k_{B}T\right)}^{2}.$$

In Equation (A7), $R(E_{F}^{0})$ is the total energy of electrons at the zero temperature, and $\frac{\pi^{2}}{6}N\left(E_{F}^{0}\right){\left(k_{B}T\right)}^{2}$ is the thermal excitation energy. Therefore, the electronic specific heat of metal can be obtained by $C_{e}=\frac{\partial U}{\partial T}$ [49]:

(A8) $$C_{e}=\frac{\pi^{2}}{3}k_{B}N\left(E_{F}^{0}\right)\left(k_{B}T\right).$$

In a 3-D bulk material, the density $N(E_{F}^{0})$ of states of electrons at the Fermi energy level is independent of shape and size. However, in an ultra-thin metal film, it can exhibit the resonantly oscillatory behavior depending on the thin film thickness.

## Appendix B. Electronic Density of States in a Thin Metal Film

We will suggest a more rigorous formulation for deriving the electronic density of states (the total number of electrons per unit volume and per unit energy), i.e., Equation (3) in a thin metal film. We have obtained the total electrons, of which the kinetic energy is lower than the Fermi energy $E_{F}$ in a thin metal film, i.e., $N_{\mathrm{tot}}\left(E_{F}\right)$ given in Equation (16). Now we shall calculate the total number, $N_{\mathrm{tot}}\left(E\right)$, of free electrons below the energy level *E* (with $E\leq E_{F}$). The result is given by

(A9) $$\begin{array}{ccc} N_{\mathrm{tot}}\left(E\right) & = & g\sum_{k}\to g\sum_{k_{z}}\frac{1}{{\left(2\pi \right)}^{2}}\int_{S}dxdy\int_{k_{⊥}}dk_{x}dk_{y}\\ & = & g\sum_{k_{z}}\frac{S}{{\left(2\pi \right)}^{2}}\pi \left[k^{2}-{\left(\frac{n\pi }{t}\right)}^{2}\right], \end{array}$$

where $k_{⊥}=\sqrt{k_{x}^{2}+k_{y}^{2}}$, $k_{z}=\frac{n\pi }{t}$ and $k^{2}=k_{⊥}^{2}+{\left(\frac{n\pi }{t}\right)}^{2}$. The maximum value of the integer *n* cannot exceed $\frac{kt}{\pi }$, i.e., $n_{\max}=\left[\frac{kt}{\pi }\right]$ (this is the integer part of $\frac{kt}{\pi }$, where *k* is lower than the Fermi wave number $k_{F}$). Then, the total number of free electrons, of which the energy is lower than *E* (with $E\leq E_{F}$) in a metal film, appears to be of the form

(A10) $$\begin{array}{c} N_{\mathrm{tot}}\left(E\right)=g\sum_{n=1}^{n_{\max}}\frac{S}{{\left(2\pi \right)}^{2}}\pi \left[k^{2}-{\left(\frac{n\pi }{t}\right)}^{2}\right]. \end{array}$$

The density of states of electrons, $N_{\mathrm{tot}}\left(E\right)$ at energy *E* (with $E\leq E_{F}$), in the present thin metal film is defined as

(A11) $$\begin{array}{ccc} N_{e}\left(E\right) & = & \frac{1}{V}\frac{\delta N_{\mathrm{tot}}\left(E\right)}{\delta E}\\ & = & \frac{1}{St}\frac{\delta }{\delta E}\left\{g\sum_{n=1}^{n_{\max}}\frac{S}{{\left(2\pi \right)}^{2}}\pi \left[k^{2}-{\left(\frac{n\pi }{t}\right)}^{2}\right]\right\}. \end{array}$$

Since $\delta E=\frac{\hbar^{2}}{2m^{*}}\delta k^{2}$, the density of states of electrons in the thin metal film given in (A11) turns out to be in the form

(A12) $$\begin{array}{c} N_{e}\left(E\right)=\frac{g}{t}\sum_{n=1}^{n_{\max}}\frac{2\pi m^{*}}{{\left(2\pi \hbar \right)}^{2}}=\frac{g}{t}\frac{2\pi m^{*}}{{\left(2\pi \hbar \right)}^{2}}n_{\max}. \end{array}$$

It should be emphasized again that the maximum integer is $n_{\max}=\left[\frac{kt}{\pi }\right]$, where *k* is lower than the Fermi wave number $k_{F}$.

Since most of the electronic properties of solid states are determined by the electrons at the Fermi energy level, we should concentrate our attention on the density of states of electrons at the Fermi surface [12,36]:

(A13) $$\begin{array}{c} N_{e}\left(E_{F}\right)=\frac{m^{*}}{\pi \hbar^{2}}\frac{\left[\frac{2t}{\lambda_{F}^{\left(\mathrm{film}\right)}}\right]}{t}, \end{array}$$

where we have used the relation $n_{\max}=\left[\frac{k_{F}t}{\pi }\right]=\left[\frac{2t}{\lambda_{F}^{\left(\mathrm{film}\right)}}\right]$, and the degree of degeneracy of spin g=2 has been substituted. If the film thickness *t* is sufficiently large, the Fermi wave number $k_{F}$ (or the Fermi wavelength $\lambda_{F}$) of the thin film can be chosen as that of 3-D bulk metal, i.e., Equation (15). If, however, the film thickness *t* is quite small (and hence, the electron quantum confinement related to the quantum mechanical de Broglie standing wave in deep potential wells plays a key role), the Fermi wave number $k_{F}$ (or the Fermi wavelength $\lambda_{F}$) of the thin film would differ much from that of the bulk metal. In this case, we should calculate the nanofilm Fermi wave number $k_{F}$ (or the Fermi wavelength $\lambda_{F}^{\left(\mathrm{film}\right)}$) by using Equations (16) and (17), which has been shown in Figure 5.

It should be pointed out that after we have studied the quantum size correction to the Fermi wavelength, we found that our result (17) is consistent with Equation (10) in Ref. [36], but the our physical understanding differs much from that in Ref. [36], where it seems that the quantum size correction to the Fermi wavelength was not taken into consideration. We have shown in this paper that when the number of atomic layers of a metal film is less than 8, the electron Fermi wavelength $\lambda_{F}$ of the metal film would be dramatically corrected by the film thickness compared with that of a metal film of more than 10 atomic layers.

## Appendix C. Comparison of the Two Cases with and without Quantum Size Correction to the Fermi Wavelength

In order to compare two cases, where the quantum size correction to the electron Fermi wavelength is taken into consideration in one case but not in the other case, let us continue with the result (23), where the Fermi wavelength is independent of the film thickness *t*. We give the ratios, $R_{1}$, defined in Equation (20), of the electron Fermi surface densities of states for the ultra-thin metal film atomic layer numbers $N_{\mathrm{layer}}=5$∼10:

(A14) $$\begin{array}{ccc} & & N_{\mathrm{layer}}=5,\;R_{1}=\frac{1.06\;\mathrm{nm}}{2\times 5\times 0.3\;\mathrm{nm}}\left[\frac{2\times 5\times 0.3\;\mathrm{nm}}{1.06\;\mathrm{nm}}\right]=0.707;\\ & & N_{\mathrm{layer}}=6,\;R_{1}=\frac{1.06\;\mathrm{nm}}{2\times 6\times 0.3\;\mathrm{nm}}\left[\frac{2\times 6\times 0.3\;\mathrm{nm}}{1.06\;\mathrm{nm}}\right]=0.883;\\ & & N_{\mathrm{layer}}=7,\;R_{1}=\frac{1.06\;\mathrm{nm}}{2\times 7\times 0.3\;\mathrm{nm}}\left[\frac{2\times 7\times 0.3\;\mathrm{nm}}{1.06\;\mathrm{nm}}\right]=0.757;\\ & & N_{\mathrm{layer}}=8,\;R_{1}=\frac{1.06\;\mathrm{nm}}{2\times 8\times 0.3\;\mathrm{nm}}\left[\frac{2\times 8\times 0.3\;\mathrm{nm}}{1.06\;\mathrm{nm}}\right]=0.883;\\ & & N_{\mathrm{layer}}=9,\;R_{1}=\frac{1.06\;\mathrm{nm}}{2\times 9\times 0.3\;\mathrm{nm}}\left[\frac{2\times 9\times 0.3\;\mathrm{nm}}{1.06\;\mathrm{nm}}\right]=0.981;\\ & & N_{\mathrm{layer}}=10,\;R_{1}=\frac{1.06\;\mathrm{nm}}{2\times 10\times 0.3\;\mathrm{nm}}\left[\frac{2\times 10\times 0.3\;\mathrm{nm}}{1.06\;\mathrm{nm}}\right]=0.883. \end{array}$$

We continue with the results (24) and (25) for the atomic layer numbers $N_{\mathrm{layer}}=1$∼4, where the Fermi wavelength depends on the film thickness *t*, i.e., we need to list the ratios $R_{2}$ defined in Equation (22) for the electron densities of states of the thin film layer numbers $N_{\mathrm{layer}}=5$∼10; specifically, the layer number-dependent Fermi wavelength $\lambda_{F}\left(N_{\mathrm{layer}}\right)$, the number $n_{\max}$ of half Fermi wavelength and the electron density of states (expressed in terms of the ratio $R_{2}$) for the cases of the film layer numbers $N_{\mathrm{layer}}=5$∼10 are given as follows:

(A15) $$\begin{array}{ccc} & & N_{\mathrm{layer}}=5,\;\lambda_{F}\left(5\right)=0.97\;\mathrm{nm},\;n_{\max}=\left[\frac{2\times 5\times 0.30\;\mathrm{nm}}{0.97\;\mathrm{nm}}\right]=3,\\ & & R_{2}=\frac{1.06\;\mathrm{nm}}{2\times 5\times 0.30\;\mathrm{nm}}\times 3=1.06;\\ & & N_{\mathrm{layer}}=6,\;\lambda_{F}\left(6\right)=0.99\;\mathrm{nm},\;n_{\max}=\left[\frac{2\times 6\times 0.30\;\mathrm{nm}}{0.99\;\mathrm{nm}}\right]=3,\\ & & R_{2}=\frac{1.06\;\mathrm{nm}}{2\times 6\times 0.30\;\mathrm{nm}}\times 3=0.883. \end{array}$$

(A16) $$\begin{array}{ccc} & & N_{\mathrm{layer}}=7,\;\lambda_{F}\left(7\right)=1.01\;\mathrm{nm},\;n_{\max}=\left[\frac{2\times 7\times 0.30\;\mathrm{nm}}{1.01\;\mathrm{nm}}\right]=4,\\ & & R_{2}=\frac{1.06\;\mathrm{nm}}{2\times 7\times 0.30\;\mathrm{nm}}\times 4=1.01;\\ & & N_{\mathrm{layer}}=8,\;\lambda_{F}\left(8\right)=1.02\;\mathrm{nm},\;n_{\max}=\left[\frac{2\times 8\times 0.30\;\mathrm{nm}}{1.02\;\mathrm{nm}}\right]=4,\\ & & R_{2}=\frac{1.06\;\mathrm{nm}}{2\times 8\times 0.30\;\mathrm{nm}}\times 4=0.883. \end{array}$$

(A17) $$\begin{array}{ccc} & & N_{\mathrm{layer}}=9,\;\lambda_{F}\left(9\right)=1.03\;\mathrm{nm},\;n_{\max}=\left[\frac{2\times 9\times 0.30\;\mathrm{nm}}{1.03\;\mathrm{nm}}\right]=5,\\ & & R_{2}=\frac{1.06\;\mathrm{nm}}{2\times 9\times 0.30\;\mathrm{nm}}\times 5=0.981;\\ & & N_{\mathrm{layer}}=10,\;\lambda_{F}\left(10\right)=1.04\;\mathrm{nm},\;n_{\max}=\left[\frac{2\times 10\times 0.30\;\mathrm{nm}}{1.04\;\mathrm{nm}}\right]=5,\\ & & R_{2}=\frac{1.06\;\mathrm{nm}}{2\times 10\times 0.30\;\mathrm{nm}}\times 5=0.883. \end{array}$$

It follows from the above results [i.e., Equations (A15)–(A17)] that the electron number density (involved in the ratio $R_{2}=\frac{N^{\left(f\right)}\left(E_{F}\right)}{N^{\left(b\right)}\left(E_{F}\right)}$) exhibits an even–odd layer oscillatory effect with the increase in the film atomic layer number $N_{\mathrm{layer}}$.
